# Modulation of Lysosomal Cl^−^ Mediates Migration and Apoptosis through the TRPML1 as a Lysosomal Cl^−^ Sensor

**DOI:** 10.3390/cells12141835

**Published:** 2023-07-12

**Authors:** Dongun Lee, Jeong Hee Hong

**Affiliations:** Department of Health Sciences & Technology, Gachon Advanced Institute for Health Sciences and Technology (GAIHST), Gachon University, 155 Gaetbeol-ro, Yeonsu-gu, Incheon 21999, Republic of Korea; sppotato1@gmail.com

**Keywords:** calcium, chloride, lysosome, TRPML1, GXXXP motif

## Abstract

Lysosomes are responsible for protein degradation and clearance in cellular recycling centers. It has been known that the lysosomal chloride level is enriched and involved in the intrinsic lysosomal function. However, the mechanism by which chloride levels can be sensed and that of the chloride-mediated lysosomal function is unknown. In this study, we verified that reduced chloride levels acutely induced lysosomal calcium release through TRPML1 and lysosomal repositioning toward the juxtanuclear region. Functionally, low chloride-induced lysosomal calcium release attenuated cellular migration. In addition, spontaneous exposure to low chloride levels dysregulated lysosomal biogenesis and subsequently induced delayed migration and promoted apoptosis. Two chloride-sensing GXXXP motifs in the TRPML1 were identified. Mutations in the GXXXP motif of TRPML1 did not affect chloride levels, and there were no changes in migratory ability. In this study, we demonstrated that the depletion of chloride induces reformation of the lysosomal calcium pool and subsequently dysregulated cancer progression, which will assist in improving therapeutic strategies for lysosomal accumulation-associated diseases or cancer cell apoptosis.

## 1. Introduction

Cl^−^ is the most abundant electrolyte and is important for its homeostatic role in living organisms. Changes in intracellular Cl^−^ concentration ([Cl^−^]_i_) participate in the determination of membrane potential and modulation of intracellular pH and cellular volume through various Cl^−^ channels and transporters [1]. Each cellular compartment possesses sophisticated Cl^−^-regulation of homeostatic cellular functions. Lysosomes contain 110 mM Cl^−^, which is more than two times higher than in the cytosol [2,3]. In recent years, high lysosomal Cl^−^ content has been noted for critical role in lysosomal functions such as degradative function and other cellular processes [3,4].

Lysosomal-mediated metabolic processes regulate cellular homeostasis and clearance responses. Abnormalities in lysosomal functions are involved in various diseases, including lysosomal storage diseases and cancers [5,6,7,8,9]. Although high lysosomal Cl^−^ content is critical, and although the primitive role of Cl^−^ in lysosomes has been addressed in the regulation of lysosomal pH, how Cl^−^ participates in lysosomal functions and which protein is involved in cellular process are not well understood. Thus, we focused on the phenomena of high luminal Cl^−^ concentrations in lysosomes and depleted Cl^−^ states-mediated cellular responses. In addition, we hypothesized that the Cl^−^ modulation-based regulation of lysosomal repositioning could suggest a potential therapeutic strategy for lysosome-based cancer targeting.

In this study, we verified that the lysosomal Ca^2+^ channel transient receptor potential (TRP) mucolipin (TRPML)1 was activated by depleted Cl^−^ and repositioned lysosomes to the juxtanuclear region. We identified, for the first time, the lysosomal Cl^−^ sensing motif in TRPML1 and verified the role of Cl^−^ as a signaling ion in lysosomal modulation. Thus, our study suggests that disturbance of Cl^−^ sensing dysregulates the lysosomal process through a TRPML1-dependent mechanism.

## 2. Materials and Methods

### 2.1. Reagents and Cell Culture

Fura-2-AM (Fura2) and BCECF-AM were purchased from TEFlabs (Cat: 0102 and 4011 B; Austin, TX, USA). Calcein-AM was purchased from Thermo (Waltham, MA, USA; Cat: C1430), and 1-Ethoxycarbonylmethyl-6-methoxyquinolinium bromide (MQAE) was purchased from Cayman Chemical (Ann Arbor, MI, USA; Cat: 19585). Pluronic acid (Pluronic F-127, 20% (*v*/*v*) in DMSO) was purchased from Invitrogen (Carlsbad, CA, USA; Cat: P-3000MP). 1,2-bis(o-aminophenoxy)ethane-N, N, N′, N′-tetraacetic acid (BAPTA)-AM was purchased from Invitrogen (Cat: B1205), and cyclopiazonic acid (CPA) was purchased from Alomone Labs (Israel; Cat: C-750). Bafilomycin-A1 (Baf) (from *Streptomyces griseus*), glycyl-Phenylalanine β-naphthylamide (GPN), histamine, 5-Nitro-2-(3-phenylpropylamino) benzoic acid (NPPB), ML-SA1, ionomycin, and U18666a were purchased from Sigma (St. Louis, MO, USA; Cat: B1793, G9512, D9754, H7125, N4779, SML0627, I9657, and 662015, respectively). Non-small cell lung cancer cell lines H1975 and A549 were obtained from American Type Culture Collection (Rockville, MD, USA; Cat: CRL-5908, Cat: CCL-185). H1975 cells were maintained in Roswell Park Memorial Institute 1640 (RPMI 1640, Invitrogen, Cat: 11875-093), and A549 cells were maintained in Dulbecco’s modified Eagle’s medium (Invitrogen, Cat: 11995-065), each containing 10% fetal bovine serum (FBS, Invitrogen; Cat: 16000-044) and 100 U/mL penicillin–streptomycin (Invitrogen, Cat: 15140122). Cells were incubated in a humidified incubator with 5% CO_2_ and 95% air at 37 °C. When the cells reached 80% confluence, they were washed with Dulbecco’s phosphate-buffered saline (Welgene, Republic of Korea; Cat: LB001-02) after the culture medium was removed. The cells were treated with trypsin/EDTA (Invitrogen; Cat: 25200-072) for 5 min. The detached cells were transferred to fresh culture dishes with coverslips for the measurement of fluorescence dye-based electrolyte changes (intracellular Ca^2+^ concentration ([Ca^2+^]_i_), [Cl^−^]_i_, intracellular pH (pH_i_), and cellular volume) and confocal microscopy, or to fresh culture dishes for western blotting, flow cytometry, and migration assays.

### 2.2. Plasmid, Small Interfering RNA (siRNA), Mutation, and DNA Transfection

The plasmids encoding GFP-tagged chloride channel (CLC)3, CLC7, transient receptor potential melastatin (TRPM)2, and TRPML1 were purchased from Origene (Rockville, MD, USA; Cat: RG221294, RG203450, RG216220, and RG201010). The siRNAs for CLC3 and CLC7 were constructed using double-promoter pFIV-H1/U6 siRNA cloning and expression vectors (System Biosciences, Palo Alto, CA, USA; Cat: SI111A-1), according to the manufacturer’s instructions. The vectors contained single-stranded DNA for human siRNA-CLC3 (sense, 5′-GCT GTG ATA GCC TTC CCT AAT CCA TAC-3′ and anti, 5′-TAT GGA TTA GGG AAG GCT ATC ACA GC-3′) and human siRNA-CLC7 (sense, 5′-GAT GAT CCA CTC AGG TTC AGT GAT TGC-3′ and anti, 5′-GCA ATC ACT GAA CCT GAG TGG ATC ATC-3′). The siRNAs of TRPML1, two pore channel (TPC)1, and TPC2 were constructed by Genolution (Seoul, Republic of Korea), and the sequences were 5′-GAUCACGUUUGACAACAAA-3′ (siTRPML1), 5′-GGAGUUACCUCGUCUUUCUUU-3′ (siTPC1-a), 5′-GCUUUGUGACCCUGUUUGAUU-3′ (siTPC1-b), 5′-GGCUUUACCGACGGUAUUAUU-3′, and 5′-CCAUCAUUGGGAUCAACUUUU-3′ (siTPC2-b). The TRPM2 and TRPML1 mutant forms were produced using the QuickChange II Site-Directed Mutagenesis Kit (Agilent Technologies, Santa Clara, CA, USA; Cat: 200523), according to the manufacturer’s instructions mentioned on the kit. Mutants of the Cl^−^-interacting motif GXXXP [10] were constructed to replace glycine and proline with alanine, which are indicated as GP/AA (G1390A/P1394A, for TRPM2), chloride binding motif (CBM)1 (G152A/P156A, for TRPML1), and CBM2 (G173A/P177A, for TRPML1), respectively. The plasmids were transfected using jetPRIME transfection reagent (Polyplus-transfection, New York, NY, USA; Cat: 114-15) according to the manufacturer’s protocol. Each plasmid was diluted in 200 μL of jetPRIME buffer, and 4 μL of the reagent was added. The mixture was incubated for 10 min at room temperature (RT) and then transferred to H1975 cells with fresh RPMI media. After 6 h of incubation, the medium was replaced with fresh medium and the cells were incubated for 24 h (CLC3, CLC7, TRPML1, CBM1, and CBM2) or 48 h (siRNA-CLC3 and siRNA-CLC7).

### 2.3. Real Time Polymerase Chain Reaction (PCR)

The mRNA expression of TRPML1 in lung cancer cells (H1975, A549 and H1299) was analyzed using real-time PCR. Total RNA was extracted using GeneAll Hybrid-R^TM^ (305-101), according to the manufacturer’s instructions. cDNA was synthesized using the AccuPower RocketScript^TM^ Cycle RT PreMix (Bionner, K-2204). RNA (1 μg) was mixed with the premix, and the PCR cycle was 4 °C (5 min) → 37 °C (90 min) → 95 °C (2 min). The synthesized cDNA was amplified using PowerUp^TM^ SYBR^TM^ Green Master Mix (Applied Biosystems, A25741). The primer sequences used were 5′-ACACCCCCAGAAGAGGAAGA-3′ (forward)/5′-CGCAGGGACTCATGAAAAAG-3′ (reverse) for TRPML1 and 5′-GACCTGACCTGCCGTCTAGAAA-3′ (forward)/5′-CCTGCTTCACCACCTTCTTGA-3′ (reverse) for GAPDH. The PCR cycles were as follows: 50 °C (2 min) → 95 °C (2 min) → [95 °C (15 s) → 57 °C (15 s) → 72 °C (1 min)] × 40 cycles → 95 °C (15 s) → 60 °C (1 min) → 95 °C (15 s). The expression level was calculated using the delta threshold cycle value and is indicated as a value relative to the control.

### 2.4. Measurement of [Ca^2+^]_i_

When the H1975 cells reached 80% confluence, they were transferred onto coverslips and incubated with 4 μM Fura2-AM and 0.05% pluronic acid in physiological salt solution (Reg) (containing 147 mM Cl^−^, Table 1) for 15 min at RT in the dark. After incubation with Fura2, the cells were washed with Reg for 5 min before measuring [Ca^2+^]_i_. Solutions with different Cl^−^ concentrations were produced as described in Table 1 (Ca^2+^ free concentration is defined as C/F). The time course of the solutions applied is represented by bars above the traces. [Ca^2+^]_i_ was determined by measuring Fura2 fluorescence using dual excitation wavelengths of 340 nm and 380 nm and an emission wavelength of 530 nm. [Ca^2+^]_i_ is represented by the fura2 fluorescence ratio (340/380). The emitted fluorescence was monitored using a CCD camera (Retiga 6000, Q-imaging, Tuscon, AZ, USA) attached to an inverted microscope (Olympus, Japan) and analyzed using a Meta Fluor system (Molecular Devices, San Jose, CA, USA). Fluorescence images, obtained at 3 s intervals, were normalized by subtracting the raw signals from the background images.

### 2.5. Measurement of [Cl^−^]_i_

The H1975 cells were transferred onto coverslips after reaching 80% confluence and incubated with 5 mM MQAE and 0.05% pluronic acid in Reg for 15 min at RT in the dark. After incubation with MQAE dye, the cells were washed with Reg for 5 min prior to measuring [Cl^−^]_i_. The time course of applied solutions is represented with bars above the traces. [Cl^−^]_i_ was determined by measuring MQAE fluorescence using an excitation wavelength of 360 nm and an emission wavelength of 530 nm. The emitted fluorescence was collected with a CCD camera (Retiga 6000) attached to an inverted microscope (Olympus) and analyzed using a Meta Fluor system (Molecular Devices). Fluorescence images were obtained at 3 s intervals.

### 2.6. Measurement of pH_i_

The pH_i_ of the H1975 cells was evaluated using BCECF fluorescence imaging. The cells were transferred onto coverslips after they had reached 80% confluence and incubated with 20 μM BCECF and the same volume of 0.05% pluronic acid to enhance loading efficacy in Reg for 15 min at RT in the dark. After incubation with BCECF dye, the cells were washed with Reg for 5 min prior to measuring pH_i_. The time course of the solutions applied is represented by the bars above the traces. pH_i_ was determined by measuring fluorescence using dual excitation wavelengths of 495 and 440 nm and an emission wavelength of 530 nm. pH_i_ is represented by the fluorescence ratio. Ratios of BCECF were converted to pH units using in situ calibration curves as described by Lee et al. [11]. The calibration curve for pH_i_ is shown in Supplementary Figure S1A. Briefly, the BCECF-loaded cells were incubated in pH 5.5 calibration solution including 20 μM nigericin (Sigma; Cat: N7143) for 5 min, and then the process was repeated at pH 6.0–8.5 (0.5 interval). The pH value was calculated from the pH calibration curve using the following equation (pH = pKa − log((R_max_ − R)/R − R_min_)) (pKa of BCECE; 6.97, R; ratio of BCECF, R_max_; maximum ratio, R_min_; minimum ratio). The emitted fluorescence was collected with a CCD camera (Retiga 6000) attached to an inverted microscope (Olympus) and analyzed using a Meta Fluor system (Molecular Devices). Fluorescence images were obtained at 3 s intervals.

### 2.7. Measurement of Cellular Volume Changes

When the confluence of H1975 cells reached 80%, they were transferred onto coverslips and incubated with 2 μM cell volume indicator calcein-AM and 0.05% pluronic acid in Reg for 15 min at RT in the dark. The calcein-loaded cells were washed with Reg for 5 min prior to measuring the volume changes. The time course of the solutions applied is represented by the bars above the traces. The cellular volume was determined by measuring the fluorescence at an excitation wavelength of 495 nm and an emission wavelength of 515 nm. The emitted fluorescence was collected with a CCD camera (Retiga 6000) attached to an inverted microscope (Olympus) and analyzed using a Meta Fluor system (Molecular Devices). Fluorescence images were obtained at 3 s intervals.

### 2.8. Immunofluorescence and Confocal Microscopy

The transferred H1975 cells onto coverslips were incubated under the indicated conditions and then fixed with 4% paraformaldehyde (PFA) in PBS for 10 min at RT. The fixed cells were washed three times with PBS and then incubated with blocking serum (0.5% BSA and 5% goat serum in PBS) for 1 h at RT to block non-specific binding sites. The blocked cells were incubated with the primary antibodies overnight at 4 °C, followed by three washes with PBS at RT. For the detection of the nuclear envelope, anti-Lamin A/C antibody (1:100 dilution in blocking serum) (Abcam, UK; Cat: ab185014) was used. Lysosomal vesicles were stained with anti-LAMP-2 mouse antibody (1:100) (Abcam; Cat: ab25631), which was detected by incubation with fluorescein isothiocyanate-tagged anti-mouse IgG antibody (green, 1:200) and rhodamine-tagged anti-mouse IgG antibody (red, 1:200) (Jackson ImmunoResearch, West Grove, PA, USA; Cat: 115-095-003 and 115-025-072) for 1 h at RT. Transcription factor EB (TFEB) was stained with anti-TFEB rabbit antibody (1:100) (Cell Signaling Technology, Danvers, MA, USA; Cat: 37785), which was detected by incubation with rhodamine-tagged anti-rabbit IgG antibody (red, 1:200) (Jackson ImmunoResearch; Cat: 111-025-003) for 1 h at RT. After incubation, the cells were washed three times, and the cover slips were mounted on a glass slide with 20 μL of Fluoromount-G containing 4′,6-diamidino-2-phenylindole (DAPI) (Electron Microscopy Sciences, Hatfield, PA, USA; Cat: 17984-24). Confocal microscopy images were obtained using an LSM 700 Zeiss confocal microscope (Carl Zeiss, Oberkochen, Germany) with ZEN software (Carl Zeiss, version 8.1). The fractional distances were calculated by the straight distance from the center of nucleus to fluorescent vesicles. The fractional distance 0 indicates the center of nucleus and the fractional distance 1 indicates plasma membrane. All image data acquisition was performed at the Cell to In Vivo Imaging Core Facility Research Center (CII, Lee Gil Ya Cancer and Diabetes Institute, Gachon University, Incheon, Republic of Korea).

### 2.9. Transmission Electron Microscopy

After incubation with Reg or 5Cl^−^ solution, H1975 cells were detached and fixed with 2% PFA and 2% glutaraldehyde in 100 mM phosphate buffer (pH 7.4) for 12 h at 4 °C. Fixed cells were washed with 100 mM phosphate buffer and post-fixed with 1% osmium tetroxide in 100 mM phosphate buffer for 2 h at RT. Thereafter, the cells were dehydrated with gradually increasing concentrations of ethanol (50–100% in water). The cells were infiltrated with propylene oxide for 10 min and embedded using a Poly/Bed 812 kit (Polysciences, Warrington, PA, USA; Cat: 08792). The specimens were sectioned at 200 nm using an ultramicrotome (EM-UCT, Leica, Teaneck, NJ, USA) and stained with toluidine blue. The specimens were sectioned at 70 nm and stained with 5% uranyl acetate for 15 min, and then with 1% lead citrate for 7 min. Transmission electron microscope (TEM) images were obtained using a transmission electron microscope (JEM-1011, JEOL, Tokyo, Japan) at an acceleration voltage of 80 kV.

### 2.10. Western Blotting

The cells incubated under the indicated conditions were dispersed in lysis buffer (Cell Signaling; 9803) containing 20 mM Tris, 150 mM NaCl, 2 mM EDTA, 1% Triton X-100, and protease inhibitor mixture in the presence (for phosphorylated form) or absence of phosphatase inhibitor mixture. The cell lysate was sonicated and centrifuged at 11,000× *g* for 15 min at 4 °C. Protein concentration was calculated using the Bradford assay kit (Bio-Rad, Hercules, CA, USA; Cat: 5000001). The obtained protein samples were incubated with sodium dodecyl sulfate (SDS) protein sample buffer, and then the samples were separated by SDS-polyacrylamide gel electrophoresis, followed by transfer onto polyvinylidene difluoride membranes soaked in methanol. The membranes were incubated for 1 h at RT with 5% non-fat milk solution in 0.05% Tris-buffered saline with 0.05% Tween-20 to block non-specific binding. The blocked membranes were incubated overnight with primary antibodies at 4 °C. CLC7 was detected using anti-CLC7 rabbit antibodies (Novusbio, Centennial, CO, USA; Cat: NBP2-30021). TRPML1 was detected using anti-TRPML1 (MCOLN1) rabbit antibody (ATLAS antibodies, Voltavagen, Sweden; Cat: HPA031763). Cleaved PARP was detected using an anti-cleaved PARP (Asp214) antibody (Cell Signaling; Cat: 9541). mTORC1s, including both total and phosphorylated forms, were detected using anti-mTOR, anti-phospho-mTOR (Ser 2448) rabbit antibody, and anti-phospho-mTOR (Ser 2481) rabbit antibodies (Cell Signaling; Cat: 2972, 2971, and 2974). TPC1 and TPC2 were detected using anti-TPCN1 and anti-TPCN2 rabbit antibodies (Alomone Labs; Cat: ACC-071 and ACC-072). After primary antibody incubation, the membranes were washed with PBS three times and then incubated with secondary antibodies: horseradish peroxidase-conjugated anti-mouse IgG and anti-rabbit IgG antibodies (Millipore, Billerica, MA, USA; Cat: AP124P and AP132P) for 1 h at RT. β-actin was detected using a horseradish peroxidase-conjugated anti-β-actin mouse antibody (Sigma; Cat: A3854). The membrane was washed three times, and the protein was detected with enhanced chemiluminescence on X-ray films.

### 2.11. Transwell Membrane Migration Assay

The dispersed H1975 cells (5 × 10^4^ cells/well) in Reg, 5 mM Cl^−^ solutions, or RPMI (with 1% FBS each) were added to the upper chamber of a 6-well transwell membrane plate (8.0 μm pore sized insert). The bottom chambers were filled with pH 7.5 or pH 6.5 RPMI (with 10% FBS and 100 U/mL penicillin–streptomycin), followed by the indicated conditions. After incubation for 3 h, the membranes were stained with DAPI (blue) or crystal violet (purple). The membranes were incubated with chilled methanol (preserved at −20 °C) for 1 min at −20 °C to fix the cells and then washed with PBS three times. For DAPI staining, the membranes were incubated with DAPI solution (1μg/mL in distilled water (DW)) for 30 min at 37 °C in the dark and then washed twice with DW. For crystal violet staining, the membranes were incubated with 0.25% crystal violet solution in DW (with 20% methanol) for 10 min at room temperature in the dark and then washed with DW six times. After washing the membranes, the media on the top was carefully removed, and DW was added to the bottom of the plate. The plates were subsequently analyzed using an LSM 700 Zeiss confocal microscope (Carl Zeiss) (for DAPI) or an inverted microscope (Olympus) with Mosaic software (Opto Science, Tokyo, Japan, version 1.6) (for crystal violet). The intensity of the obtained images was measured using the Meta Morph system (Molecular Devices).

### 2.12. Flow Cytometry

To analyze cell viability, H1975 cells were treated with Reg and 5 mM Cl^−^ solution under the indicated conditions (0.5, 1, 6, 12, and 24 h). Thereafter, the cells were washed with annexin V binding buffer (50 mM HEPES, 700 mM NaCl, 12.5 mM CaCl_2_, pH 7.4) and suspended in 100 μL of annexin V binding buffer. A single-cell suspension was treated with Pacific Blue-conjugated annexin V (Thermo; Cat: A35122) and propidium iodide (PI, Thermo; Cat: P1304MP) for 15 min at room temperature in the dark. The negative control was incubated with annexin V-binding buffer without annexin V and PI. After incubation, 500 μL of annexin V buffer was added to the samples, and the samples were analyzed at 410 nm excitation with a 455 nm band-pass filter to detect Pacific blue and 535 nm excitation with a 620 nm band-pass filter to detect PI. The percentage of late apoptosis was calculated by analyzing the first quadrant of the cell population, which was determined using annexin V and PI fluorescence.

### 2.13. pHRodo Staining

The transferred H1975 cells onto coverslips were incubated with Reg and 5 mM Cl^−^ solution for 30 min at 37 °C and then fixed with 4% PFA in PBS for 10 min at RT. The fixed cells were washed with PBS three times, and then treated with a pHRodo Green AM Intracellular pH Indicator (Invitrogen; Cat: P35373) in Reg for 30 min at 37 °C according to the manufacturer’s instructions. After incubation, for the measurement of pH calibration, the cells were incubated with different pH values of Reg (4.5, 5.5, 6.5, and 7.5). The calibration curve of pHRodo is shown in Supplementary Figure S1B. The coverslips were mounted on glass slides, and images were obtained using an LSM 700 Zeiss confocal microscope (Carl Zeiss, Germany) with ZEN software (Carl Zeiss).

### 2.14. Statistical Analyses

Data from the indicated number of experiments were expressed as mean ± standard error of the mean (SEM). Statistical significance was determined by analysis of variance for each experiment (* *p* < 0.05, ** *p* < 0.01, *** *p* < 0.001), which was analyzed using ANOVA (followed by a Newman-Keuls multiple comparison test).

## 3. Results

### 3.1. Reduced [Cl^−^]_e_ Induced Lysosomal Ca^2+^ Release

**Figure 1:**
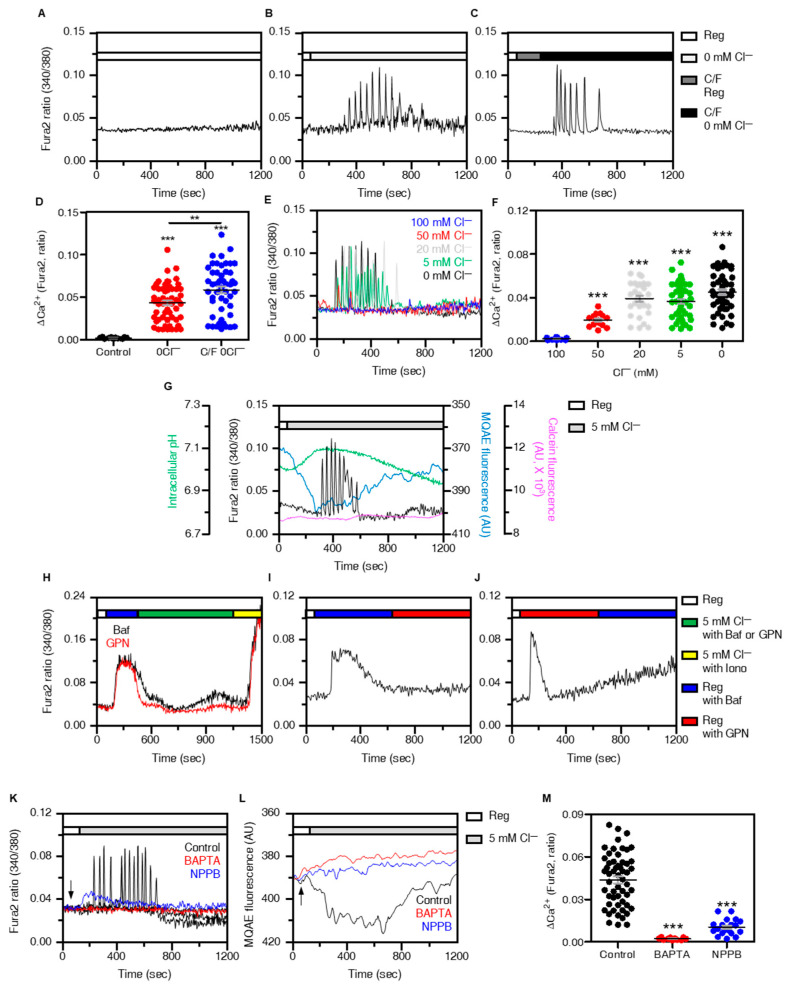
Reduced [Cl^−^]_e_ induced lysosomal Ca^2+^ release. (**A**–**C**) Changes in [Ca^2+^]_i_ in H1975 cells under the indicated conditions. (**D**) The dot plots are presented as means ± SEMs of the relative changes of [Ca^2+^]_i_ spikes (ΔCa^2+^, n = 11, n = 57, n = 50, ** *p* < 0.01, *** *p* < 0.0001). (**E**) Changes in [Ca^2+^]_i_ in H1975 cells with various range of Cl^−^ concentration solutions from 0 to 100 mM. (**F**) The dot plots are presented as means ± SEMs of the relative changes of [Ca^2+^]_i_ spikes (ΔCa^2+^, n = 13, n = 15, n = 29, n = 47, n = 53, *** *p* < 0.0001). (**G**) Combined traces of intracellular pH, Ca^2+^ (Fura2), Cl^−^ (MQAE), and cellular volume (calcein) in presence of low Cl^−^ (5Cl^−^). (**H**) Changes in [Ca^2+^]_i_ in H1975 cells with 5Cl^−^ in the presence of Baf (2 μM, black) or GPN (50 μM, red) which were followed by treatment of ionomycin (50 μM). (**I**,**J**) Changes in [Ca^2+^]_i_ in H1975 cells induced by 2 μM Baf and 50 μM. (**K**,**L**) Changes in [Ca^2+^]_i_ (**K**) and [Cl^−^]_i_ (**L**) with 5Cl^−^ in the presence of BAPTA-AM (10 μM, red) or NPPB (50 μM, blue). (**M**) The dot plots are presented as means ± SEMs of the relative changes of [Ca^2+^]_i_ spikes (ΔCa^2+^, n = 55, n = 11, n = 20, *** *p* < 0.0001). Time course of each reagent stimulation is represented with arrows. Each fluorescence measurement proceeded under the conditions indicated on the bars on the right or upper side. The number of dot plots mean the number of responding cells which have [Ca^2+^]_i_ signals.

Although [Cl^−^]_i_ is involved in homeostatic functions of cellular system, the role of Cl^−^ as a signaling ion is poorly understood. In this study, we found that stimulation with low Cl^−^ concentrations enhanced Ca^2+^ signaling, whereas there were no changes for [Ca^2+^]_i_ signal in Reg (Figure 1A,B). Decreasing Cl^−^ concentration in extracellular media by using 0 mM Cl^−^ solution induced Ca^2+^ oscillation for a short period of time (approximately 5 min) in H1975 cells (Figure 1B–D) suggested that Ca^2+^ oscillation occurred in intracellular compartments. Exposure of decreased [Cl^−^]_e_, ranging from 0 to 20 mM, induced Ca^2+^ signaling in H1975 cells (Figure 1E,F). The 0 mM Cl^−^ solution did not exist physiologically. Thus, the following studies were performed with 5 mM Cl^−^ as the low Cl^−^ condition. As shown in Figure 1G, we measured changes of [Ca^2+^]_i_ signal, intracellular pH, [Cl^−^]_i_, and cellular volume in the presence of low Cl^−^ media. [Cl^−^]_i_ decreased before starting to reveal the [Ca^2+^]_i_ oscillation in low Cl^−^ extracellular media (Figure 1G). The intracellular pH (pH_i_) increased before increasing [Cl^−^]_i_ and [Ca^2+^]_i_, and then gradually decreased (Figure 1G). The cellular volume with calcein-AM fluorescence-mediated measurement did not change in H1975 cells in low Cl^−^ extracellular media (Figure 1G). In other words, the changes of [Ca^2+^]_i_ are induced by the decrease in [Cl^−^]_i_ with an increase in pH_i_ regardless of osmotic pressure. To demonstrate the source of low Cl^−^-mediated [Ca^2+^]_i_ oscillations, we hypothesized that intracellular compartment lysosomes, which possess ion channels such as CLCs, TPC, and TRPMLs, transfer Cl^−^, and Ca^2+^ [5,12]. To deplete lysosomal Ca^2+^, cells were treated with lysosomal Ca^2+^ exhausting agents, V-ATPase inhibitor bafilomycin (Baf) [13], or selective lysosomal depletion agent GPN [14]. Low Cl^−^-mediated Ca^2+^ signaling did not increase after treatment with Baf or GPN (Figure 1H). Ca^2+^ ionophore ionomycin-evoked Ca^2+^ signaling reflected that Baf or GPN did not deplete whole intracellular Ca^2+^ store (Figure 1H). Baf/GPN-induced Ca^2+^ signaling induced the exhaustion of lysosomal Ca^2+^; therefore, no changes in [Ca^2+^]_i_ were observed after GPN/Baf stimulation (Figure 1I,J). In addition, treatment with U18666A, which depletes lysosomal Ca^2+^ without affecting V-ATPase [15], decreased low Cl^−^-mediated Ca^2+^ signaling (Supplementary Figure S2A,B). Ca^2+^ chelator BAPTA-AM and Cl^−^ channel blocker NPPB attenuated [Ca^2+^]_i_ and [Cl^−^]_i_ signaling in H1975 cells (Figure 1K–M). To determine the low Cl^−^-mediated Ca^2+^ source, treatment with CPA or histamine, which induces the Ca^2+^ release of endoplasmic reticulum, was conducted, and had no effect on low Cl^−^-mediated Ca^2+^ signaling (Supplementary Figure S2C–F). These results demonstrate that the low Cl^−^-mediated [Ca^2+^]_i_ oscillation was mediated from the lysosomal Ca^2+^ source.

### 3.2. Low Cl^−^-Induced Lysosomal Reposition and Deterioration of Migration

**Figure 2:**
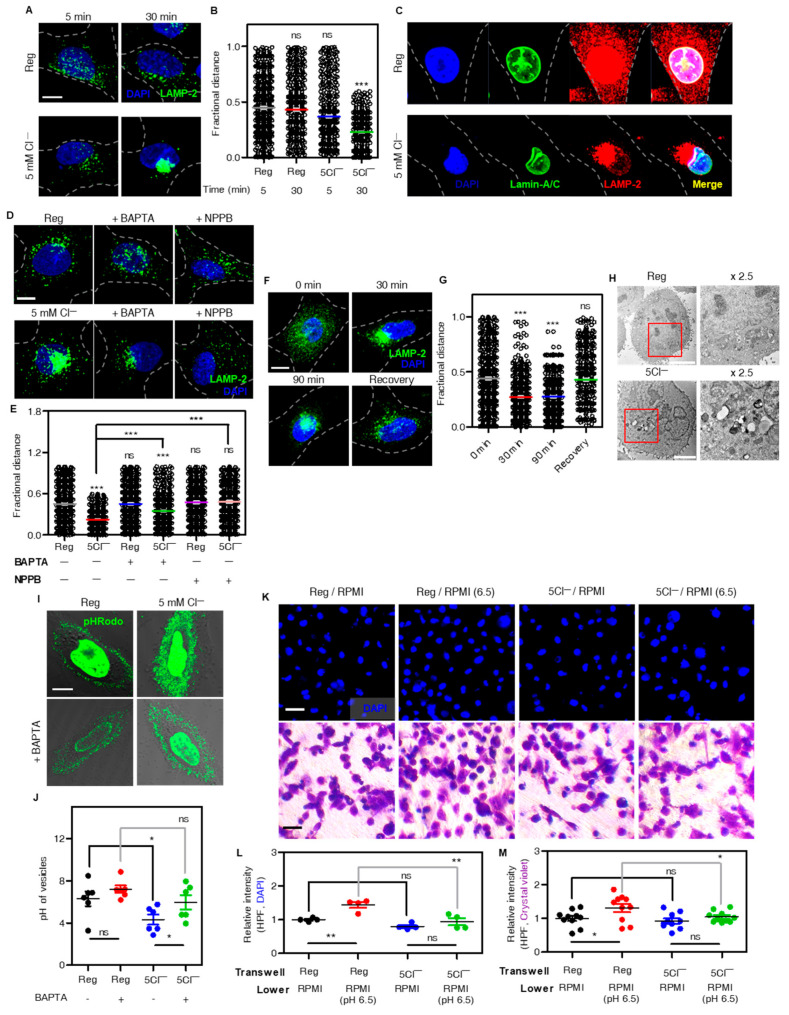
Low Cl^−^-induced lysosomal reposition and deterioration of migration. (**A**) Images of immunofluorescence staining of LAMP−2 protein (green) and DAPI (blue) in H1975 cells which were incubated under the indicated conditions. The scale (white) represents 10 μm. (**B**) The dot plots are presented as means ± SEMs of the fractional distance. Each value was obtained from n = 5 independent images (*** *p* < 0.0001, ns; non-significance). (**C**) Confocal images stained for nuclear envelop (Lamin-A/C, green), LAMP-2 protein (red), and DAPI (blue) in H1975 cells which incubated with Reg and 5Cl^−^ solutions for 30 min. The scale (white) represents 10 μm. (**D**) Confocal images of the immunofluorescence staining of LAMP−2 (green) and DAPI (blue), which were incubated with Reg and 5Cl^−^ solution for 30 min in the presence or absence of BAPTA−AM and NPPB. The scale (white) is 10 μm. (**E**) The dot plots are presented as means ± SEMs of the fractional distance. Each value was obtained from n = 5 independent images (*** *p* < 0.0001, ns; non-significance). (**F**) Immunofluorescence images stained for LAMP−2 (green) and DAPI (blue) incubated under the indicated conditions (5Cl^−^ solution for 30 min followed by incubation of media for 60 min, called Recovery). The scale (white) represents 10 μm. (**G**) The dot plots are presented as means ± SEMs of the fractional distance. Each value was obtained from n = 5 independent images (*** *p* < 0.0001, ns; non-significance). (**H**) Transmission electron microscopy images for intracellular vesicles in H1975 cells with Reg or 5Cl^−^ solutions. The scale bar shows 5 μm. (**I**) Confocal microscopy images stained with pHRodo dye which were incubated under the indicated conditions. The scale (white) represents 10 μm. (**J**) The dot plots are presented as means ± SEMs of the pH value of intracellular vesicles (n = 6, * *p* < 0.05, ns; non-significant). (**K**) Images of transwell migration assay with DAPI (blue) or crystal violet (purple) incubated under the indicated conditions in H1975 cells. The scale (white for DAPI and black for crystal violet) represents 50 μm. (**L**) The dot plots are presented as means ± SEMs of the relative intensity of DAPI (n = 4, ** *p* < 0.01, ns; non-significance). (**M**) The dot plots are presented as means ± SEMs of the relative intensity of crystal violet (n = 10, * *p* < 0.05, ns; non-significance).

Because lysosomal Ca^2+^ release induces lysosomal movement toward the juxtanuclear region [16], we determine the low Cl^−^-mediated lysosomal reposition. Attenuated [Cl^−^]_e_ and subsequent [Ca^2+^]_i_ increase induced lysosomal repositioning with the lysosomal membrane protein marker LAMP-2 staining in the perinuclear region (Figure 2A,B). The perinuclear clustering lysosome was located in a nuclear dent and stained with the nuclear envelope marker Lamin-A/C (Figure 2C). Treatment with BAPTA-AM and NPPB blocked lysosomal repositioning in low Cl^−^-exposed H1975 cells (Figure 2D,E). Low Cl^−^-mediated lysosomal repositioning was dispersed into the cytosol through re-incubation with cell culture media, called recovery (Figure 2F,G). These results demonstrated that low Cl^−^-mediated [Ca^2+^]_i_ oscillation induced lysosomal repositioning toward the perinuclear region with a reversible process. The acidic pH of lysosomes maintains hydrolases activity in lysosomes and induces formation of lysosome-autophagosome fusion [17,18]. TEM images confirmed the low Cl^−^-induced movement of cellular vesicles toward the perinuclear region (Figure 2H). Incubation of low Cl^−^ acidified lysosomal vesicles was indicated by an enhanced pHRodo fluorescence signal, and this was inhibited by treatment with Ca^2+^ chelator BAPTA-AM (Figure 2I,J). Next, we determined the functional role of low Cl^−^ media in cancer cells. Lysosomal Ca^2+^ release and an acidic extracellular environment provided favorable intrinsic and extrinsic circumstances for the migration of cancer cells [19,20,21]. Cancer cells were exposed to an acidic environment, and their migration of cancer cells increased in acidic pH_i_ [19]. The migration assay with both DAPI and crystal violet showed that H1975 cell migration were enhanced by acidic stimulation at pH 6.5 from the lower plate, whereas [Cl^−^]_e_ depletion surrounding H1975 decreased cell migration in the acidic lower plate (Figure 2K–M). These results indicated that lysosomal Ca^2+^ release induced lysosomal movement and low Cl^−^ stimulation attenuated cellular migration.

### 3.3. TRPML1 Sensed [Cl^−^]_i_ through GXXXP Motif to Increase Lysosomal Ca^2+^ Release

**Figure 3:**
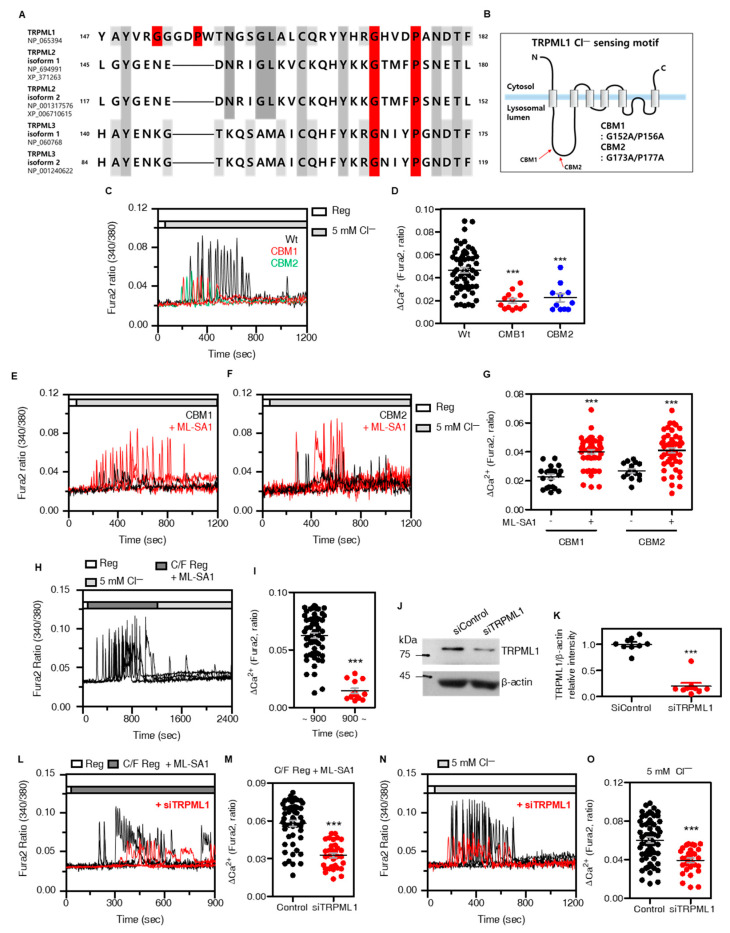
TRPML1 sensed [Cl^—^]_i_ through GXXXP motif to increase lysosomal Ca^2+^ release. (**A**) Amino acid sequences of TRPML1, TRPML2, and TRPML3 isoforms marked with predicted GXXXP Cl^—^ sensing motif (red). (**B**) schematic illustration of TRPML1 structure marked with mutation sites of GXXXP motif (CBM1 and CBM2). (**C**) Changes in [Ca^2+^]_i_ with 5Cl^—^ stimulation which transfected TRPML1 wild type (Wt, black), CBM1 (red), and CBM2 (blue). (**D**) The dot plots are presented as means ± SEMs of the relative changes of [Ca^2+^]_i_ spikes (ΔCa^2+^, n = 59, n = 13, n = 11, *** *p* < 0.0001). (**E**,**F**) Changes in [Ca^2+^]_i_ with 5Cl^—^ stimulation in the presence (red) or absence (black) of ML-SA1 (50 μM), which transfected CBM1 (**F**) and CBM2 (**G**). (**G**) The dot plots are presented as means ± SEMs of the relative changes of [Ca^2+^]_i_ spikes (ΔCa^2+^, n = 52, n = 20, n = 52, n = 15, *** *p* < 0.0001). (**H**) Changes in [Ca^2+^]_i_ in H1975 cells with the treatment of ML-SA1 before 5Cl^—^ stimulation. (**I**) The dot plots are presented as means ± SEMs of the relative changes of [Ca^2+^]_i_ spikes (ΔCa^2+^, n = 57, n = 13, *** *p* < 0.0001). ~900 (black dots); the ΔCa^2+^ from 0 s to 900 s, 900~ (red dots); the ΔCa^2+^ from 900 s to the end. Each fluorescence measurement proceeded under the conditions indicated on the bars on the upper side. (**J**) Western blotting assay for TRPML1 in siTRPML1-transfected H1975 cells. (**K**) The dot plots are presented as means ± SEMs of the protein band of TRPML1 normalized to β-actin (n = 9, *** *p* < 0.0001). (**L**) Changes in [Ca^2+^]_i_ in H1975 cells with ML-SA1 in free of [Ca^2+^]_ex_ with (red) or without (black) siRNA−TRPML1. (**M**) The dot plots are presented as means ± SEMs of the relative changes of [Ca^2+^]_i_ spikes (ΔCa^2+^, n = 52, n = 32, *** *p* < 0.0001). (**N**) Changes in [Ca^2+^]_i_ in H1975 cells in 5Cl^—^ stimulation with (red) or without (black) SiRNA-TRPML1. (**O**) The dot plots are presented as means ± SEMs of the relative changes of [Ca^2+^]_i_ spikes (ΔCa^2+^, n = 60, n = 30, *** *p* < 0.0001).

TRPML1-mediated Ca^2+^ release is important for lysosomal fusion in the perinuclear region [16]. Whether the changes in Cl^−^ act as a boosting signal on TRPML1 activation is still unclear. Thus, we evaluated whether TRPML1 possesses a GXXXP motif as a [Cl^−^]_i_ sensing motif, such as in CLC [10,22], Slc26a6 [23], and NBCe1-B [24]. Analysis of amino acid sequences showed that two GXXXP motifs were present in TRPML1. We constructed GXXXP-mutated TRPML1 mutants, named CBM1 and CBM2 (Figure 3A,B), and determined whether 5 mM [Cl^−^]_e_ solution increased Ca^2+^ signaling in TRPML1 CBM1- and CBM2-transfected H1975 cells. To determine the role of TRPML1 on the low Cl^−^-mediated Ca^2+^ release, cells were stimulated with the synthetic TRPML1 agonist ML-SA1 [25] in TRPML1 wild type and mutants (CBM1, and CBM2)-transfected H1975 cells. Although ML-SA1 activates all TRPML channels, TRPML1~3 [26], TRPML2, and TRPML3 reveal restricted cytosolic distribution not in late endosomes and lysosomes [27]. Thus, ML-SA1 was treated to activate TRPML1-mediated lysosomal Ca^2+^ release. Overexpressed Cl^−^-binding motif mutants, CBM1 or CBM2, revealed that low Cl^−^-mediated Ca^2+^ signaling was reduced (Figure 3C,D). Lysosomal Ca^2+^ channels also include NAADP-induced TPCs [28]. The GXXXP motifs of the TPCs were analyzed in the cytosolic area (Supplementary Figure S3A). The expression of TPC1 was relatively low; therefore, we developed siRNA-TPC2 (siTPC2-a and siTPC2-b) (Supplementary Figure S3B). Knockdown of TPC2 had no effect on low Cl^−^-mediated Ca^2+^ signaling (Supplementary Figure S3C). In addition, the GXXXP-mutated TRPM2, GP/AA mutant, had no effect on low Cl^−^-mediated Ca^2+^ signaling (Supplementary Figure S3D–F). Additionally, we confirmed the protein expression of TRPML1 mutants (Supplementary Figure S4A,B) and the localization of these mutants was in lysosome as co-localized with LAMP-2 (Supplementary Figure S4C–E). To confirm the channel function of the TRPML1 mutants, ML-SA1 was stimulated in the two mutants. The [Ca^2+^]_i_ was induced in only 5 mM [Cl^−^]_e_ solution, whereas both CBM1 and CBM2 were released Ca^2+^ upon treatment with ML-SA1, (Figure 3E–G). However, the lysosomal pH of all TRPML1 constructs, wild-type and mutant, did not change (Supplementary Figure S4F–I). The ML-SA1 treatment mediated lysosomal Ca^2+^ release in Ca^2+^ free (C/F) media, whereas no Ca^2+^ release occurred in presence of low Cl^−^ (Figure 3H,I). To confirm whether the low Cl^−^-mediated Ca^2+^ signaling is mediated by TRPML1, we developed siRNA-TRPML1 and evaluated the expression efficacy of siRNA (Figure 3J,K), which decreases ML-SA1-induced Ca^2+^ signaling (Figure 3L–O). The low Cl^−^-mediated lysosomal Ca^2+^ signaling was decreased by siTRPML1 treatment (Figure 3L–O). In addition, knock-down of TRPML1 decreased low Cl^−^-induced Ca^2+^ signaling in the depletion of [Ca^2+^]_ex_ (Supplementary Figure S5). These results indicated that the GXXXP motif is essential for the Cl^−^ sensing of TRPML1 to release lysosomal Ca^2+^.

### 3.4. Disturbed GXXXP Motif of TRPML1 Did Not Reduce Cellular Migration in Low Cl^−^ Stimulation

**Figure 4:**
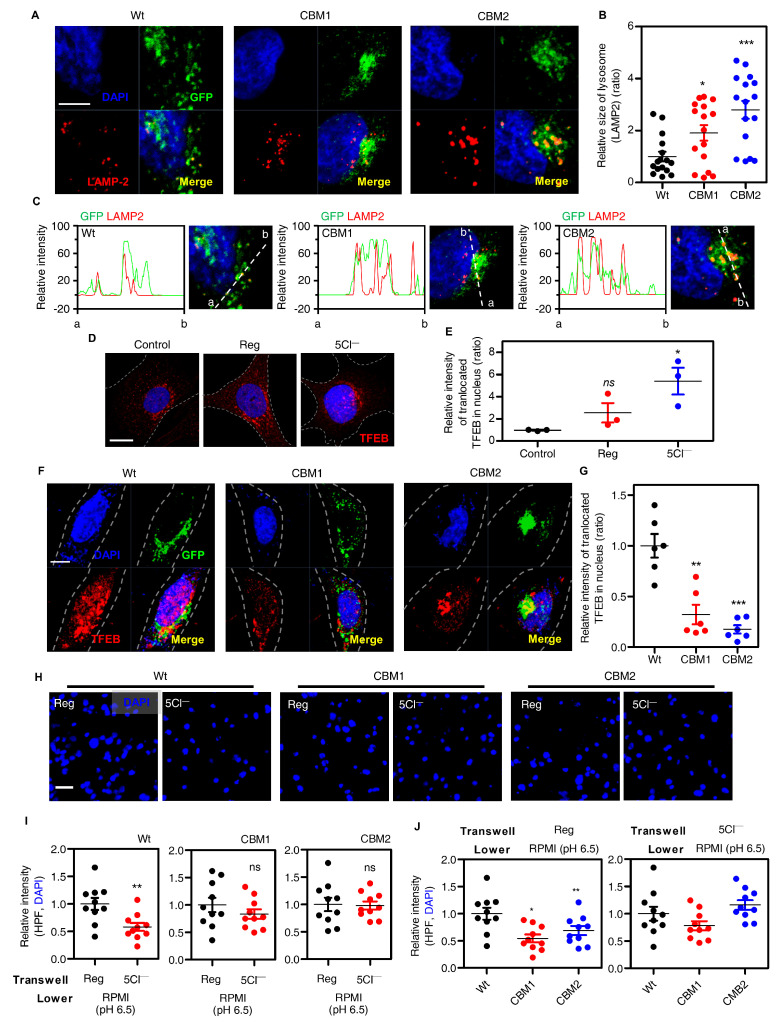
Disturbed GXXXP motif of TRPML1 did not reduce cellular migration in low Cl^−^ stimulation. (**A**) Confocal images of LAMP-2 (red) and DAPI (blue) with transfection of GFP-tagged TRPML1 Wt, CBM1, and CBM2 (green). The scale (white) represents 5 μm. (**B**) The dot plots are presented as means ± SEMs of the relative area of LAMP−2 (n = 15, * *p* < 0.05, *** *p* < 0.001). (**C**) The fluorescence intensity profiles represented enlargement of lysosomes in GFP (green) and Rhodamine (red) signals of the indicated regions of panel A. The dotted line (white) represents region of intensity profiles between point ‘a’ and point ‘b’. (**D**) Confocal images of TFEB (red) and DAPI (blue) in 5Cl^−^ stimulation. The scale (white) represents 10 μm. (**E**) The dotted plots are presented as means ± SEMs of the relative area of LAMP−2 (n = 12, * *p* < 0.05). (**F**) Confocal images of immunofluorescence staining of TFEB proteins (red) and DAPI (blue) with transfection of GFP-tagged TRPML1 Wt, CBM1, and CBM2 (green). The scale (white) represents 10 μm. (**G**) The dot plots are presented as means ± SEMs of the relative intensity of translocated TFEB in nucleus (n = 6, ** *p* < 0.01, *** *p* < 0.001). (**H**) Images of transwell migration assay with DAPI (blue) or crystal violet (purple) in TRPML1 Wt, CBM1, and CBM2-transfected cells under the indicated conditions. The scale (white) represents 10 μm. (**I**,**J**) The dot plots are presented as means ± SEMs of the relative intensity of DAPI and crystal violet (n = 8, * *p* < 0.05, ** *p* < 0.01, ns; non-significant). (**I**) The dot plots are coupled with treatment of solution. (**J**) The dot plots are coupled with TRPML1 clones, Wt, CBM1, and CBM2.

We verified the effect of TRPML1 mutants on lysosomal appearance and cellular migration. Confocal images of TRPML1 mutant-transfected H1975 cells showed enlarged lysosomes, compared to TRPML1 wild-type transfected cells (Figure 4A,B). The fluorescence intensity profiles also showed enlarged lysosomal fraction in the two mutants (Figure 4C). Transcription factor EB (TFEB) is a master regulator that translocates to the nucleus and is involved in lysosomal biogenesis [29]. In the amino acid starvation state, TFEB translocates to the nucleus and coordinates lysosomal and autophagic gene expression [30]. We compared the changes in TFEB expression in culture media, Reg, and low Cl^−^ solution. Enhanced TFEB translocation into the nucleus was observed at low Cl^−^ media (Figure 4D,E). The CBM1- and CBM2-transfected cells showed less translocation of TFEB as compared to that in TRPML1 wild-type transfected cells (Figure 4F,G). These results indicated that the disturbed Cl^−^ sensing motif of TRPML1 mediates lysosomal dysfunction, such as enlarged lysosomes and reduced TFEB translocation. We examined whether the disturbed Cl^−^ sensing ability of TRPML1 affected cellular migration. Cellular migration in the presence of acidic pH_e_ and treatment with ML-SA1 was enhanced and no additive effect on cellular migration was observed in either ML-SA1 or acidic conditions (Supplementary Figure S6A,B). The TRPML1 mutants CBM1 and CBM2 showed reduced migration in Reg compared to wild type, whereas there were no changes in migration in low Cl^−^ solutions compared to wild type (Figure 4H–J and Supplementary Figure S6C,D). These results indicate that the mutated GXXXP motif disturbs the Cl^−^sensing ability of TRPML1 and reduces the cellular migratory ability.

### 3.5. CLC7 Is Involved in the Lysosomal Cl^−^ Transfer and Expression of Lysosomal Proteins

**Figure 5:**
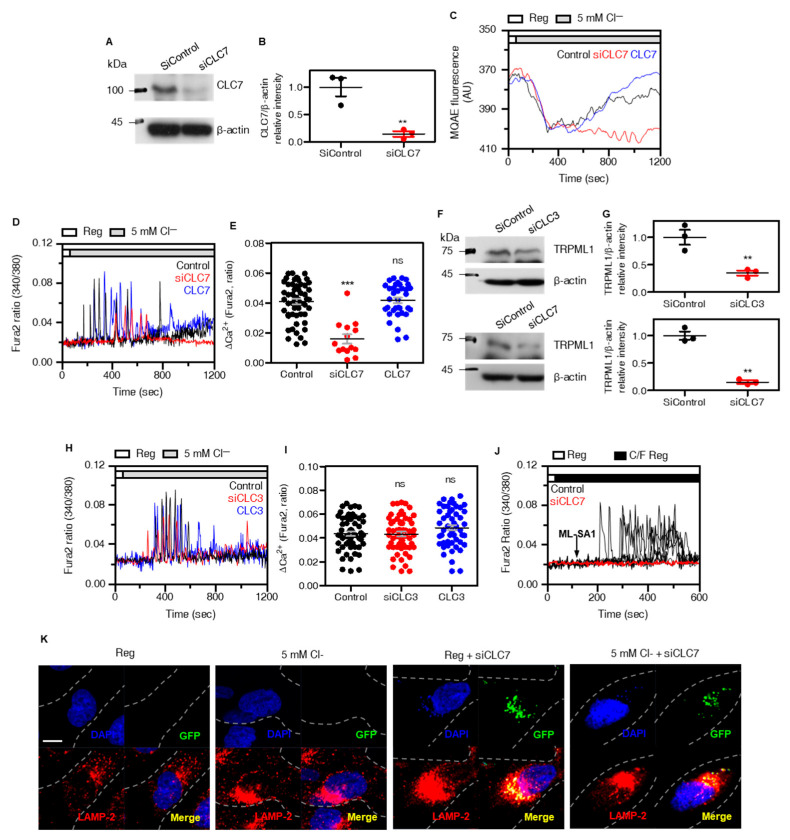
CLC7 is involved in the lysosomal Cl^−^ transfer and expression of lysosomal proteins. (**A**) Western blotting analysis of CLC7 transfected with siCLC7. Β-actin was used as a loading control. (**B**) The dot plots are presented as means ± SEMs of the protein band of CLC7 normalized to β-actin (n = 3, ** *p* < 0.01). (**C**) MQAE traces for Cl^−^ movement which silenced CLC7 (red) and over-expressed CLC7 (blue) in 5Cl^−^ stimulation. (**D**) Changes in [Ca^2+^]_I_ with 5Cl^−^ stimulation which silenced CLC7 (red) and over-expressed CLC7 (blue). (**E**) The dot plots are presented as means ± SEMs of the relative changes of [CI^2+^]_i_ spikes (ΔCa^2+^, n = 55, n = 14, n = 35, *** *p* < 0.0001, ns; non-significance). (**F**) Western blotting analysis of TRPML1 transfected with siCLC3 and siCLC7. (**G**) The dot plots are presented as means ± SEMs of the protein band of TRPML1 normalized to β-actin (n = 3, ** *p* < 0.01). (**H**) Changes in [Ia^2+^]_i_ with low Cl^−^ stimulation which silenced CLC3 (red) and over-expressed CLC3 (blue). (**I**) The dot plots are presented as means ± SEMs of the relative changes of [Ca^2+^]_i_ spikes (ΔCa^2+^, n = 54, n = 58, n = 50, ns; non-significance). (**J**) Changes in [Ca^2+^]_i_ in H1975 cells with 5Cl^−^ stimulation and treatment of ML-SA1 (50 μM) which transfected with siCLC7. (**K**) Immunofluorescence images stained for GFP (green), LAMP-2 (red), and DAPI (blue) which were incubated under the indicated conditions (Reg or 5Cl^−^ solution for 30 min) in GFP-tagged siCLC7-transfected cells. The scale (white) represents 10 μm.

Since CLC7 is expressed in late endosomes and lysosomes and is proposed to be the lysosomal Cl^−^ channel [31,32,33], we evaluated whether depleted lysosomal Cl^−^ channel CLC7 affects TRPML1-mediated lysosomal Ca^2+^ release. CLC7 knockdown with siRNAs was evaluated based on CLC7 protein expression (Figure 5A,B). Silencing of CLC7 with siRNAs suppressed lysosomal Cl^−^-induced release of lysosomal Cl^−^ into the cytosol (Figure 5C) and reduced low Cl^−^-mediated lysosomal Ca^2+^ release (red traces and dots, Figure 5D,E). Whereas overexpression of CLC7, we observed no changes compared to control in Cl^−^ and Ca^2+^ measurement (blue traces and dots, Figure 5D,E). To confirm the low Cl^−^-mediated Ca^2+^ release through the involvement of CLC7, the role of CLC3, which is broadly expressed, was determined. In contrast to knockdown of CLC7, siRNA-CLC3 has no effect on low Cl^−^-mediated Ca^2+^ release (Figure 5F–I). CLC3 knockdown with siRNAs was evaluated based on CLC3 protein expression (Supplementary Figure S7A). Knockdown of CLC7 reduced TRPML1 expression (Figure 5F,G and Supplementary Figure S7B) and lysosomal protein mTORC1 expression (total form and two types of phosphorylated form S2448, S2481 [34]) (Supplementary Figure S7B). Knockdown of TRPML1 reduced mTORC1 expression but did not reduce CLC7 expression (Supplementary Figure S7C). Knockdown of CLC3 also attenuated TRPML1 expression (Figure 5F,G). However, low Cl^−^-mediated Ca^2+^ increase was not inhibited by siCLC3 (Figure 5H,I). This result indicated that TRPML1 still existed in knockdown of CLC3. To confirm the reduced TRPML1 expression in siRNA-CLC7 cells, cells were stimulated with ML-SA1 in C/F media. Knockdown of CLC7 suppresses ML-SA1-activated Ca^2+^ signaling (Figure 5J), suggesting that CLC7 is important for lysosomal Cl^−^ regulation and lysosomal protein expression such as TRPML1. To confirm CLC7 as a lysosomal Cl^−^ path, cells were investigated for lysosomal localization in depleted CLC7. Lysosomal localization in CLC7-depleted H1975 cells was observed in the juxtanuclear region, regardless of Reg or low Cl^−^ stimulation (Figure 5K). These results indicate that CLC7 is involved in the lysosomal Cl^−^ transfer, and knockdown of CLC7 attenuates lysosomal protein expression, such as TRPML1 and mTORC1.

### 3.6. Extended Low Cl^−^ Treatment Induces Lysosomal Depletion and Apoptosis

**Figure 6:**
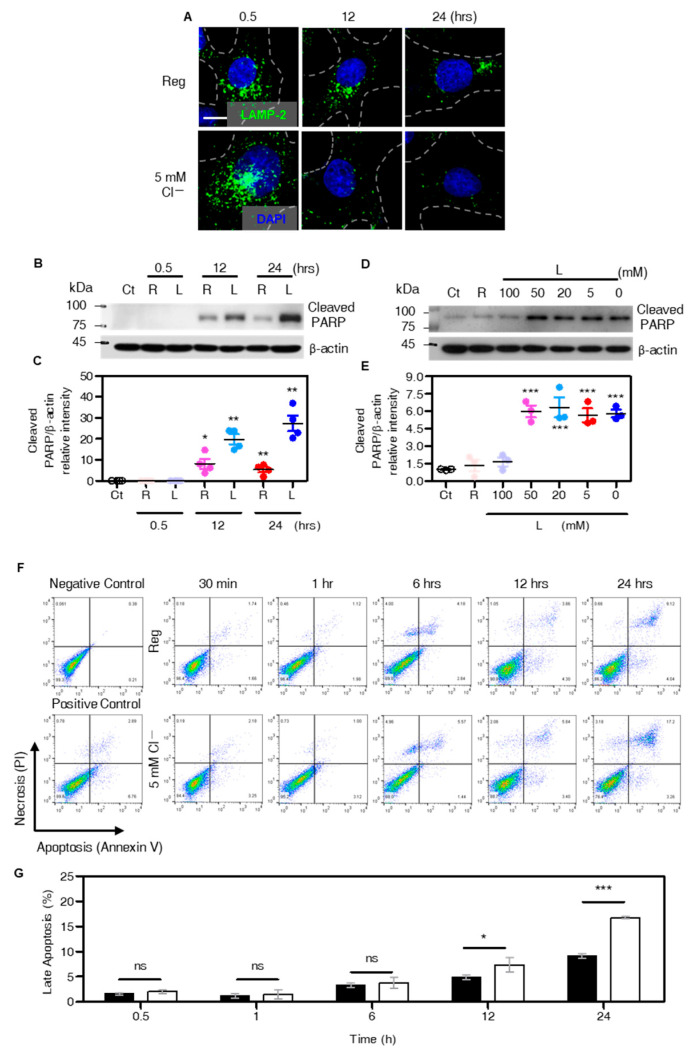
Extended low Cl^−^ treatment induces lysosomal depletion and apoptosis. (**A**) Immunofluorescence images stained with LAMP-2 (green) and DAPI (blue) which were incubated under the indicated conditions (Reg or 5Cl^−^ solution for 30 min, 12 h, and 24 h). The scale (white) represents 10 μm. (**B**) Western blotting analysis of cleaved PARP and β-actin in H1975 cells incubated with Reg (R) and 5Cl^−^ solution (L) during long-term application of the indicated time. (**C**) The dot plots are presented as means ± SEMs of the protein band of cleaved PARP normalized to β-actin (n = 4, * *p* < 0.05, ** *p* < 0.01). (**D**) Western blotting analysis of cleaved PARP and β-actin in H1975 cells incubated with Reg (R) and dose-dependent low Cl^−^ solution (L, 100 mM to 0 mM) for 24 h. (**E**) The dot plots are presented as means ± SEMs of the protein band of cleaved PARP normalized to β-actin (n = 3, *** *p* < 0.001). (**F**) FACS analysis of apoptosis using Pacific blue-conjugated Annexin V and propidium iodide (PI). Cells were treated with Reg or 5 mM Cl^−^ solution at indicated time. (**G**) The bars show the percentage of cells on late apoptosis (n = 3, * *p* < 0.05, *** *p* < 0.0001, ns; non-significant).

Extended treatment of low Cl^−^, for which even low Cl^−^-induced lysosomal Ca^2+^ signaling is over, decreased migration of H1975 cells. In the long-term application for 12 h and 24 h, lysosomes were depleted which stained with LAMP-2 in comparison with 30 min incubation is presented in Figure 6A. To determine the effect of lysosomal depletion, apoptotic signals was determined. Cleaved PARP, a hallmark of apoptosis [35,36,37], was observed in a time-dependent manner after long-term application of low Cl^−^ solution (Figure 6B,C). The expression of cleaved PARP was enhanced in a Cl^−^ dose-dependent manner (0 mM to 50 mM) in accordance with lysosomal Ca^2+^ release, (Figure 6D,E). Incubation with low Cl^−^ solution for 24 h increased late apoptosis of H1975 cells in comparison with that of the Reg-treated cells (Figure 6F,G). These results indicate that long-term treatment of low Cl^−^ induced lysosomal depletion and apoptosis.

### 3.7. The Similar Effect of Low Cl^−^ on Other Lung Cancer Cell A549

**Figure 7:**
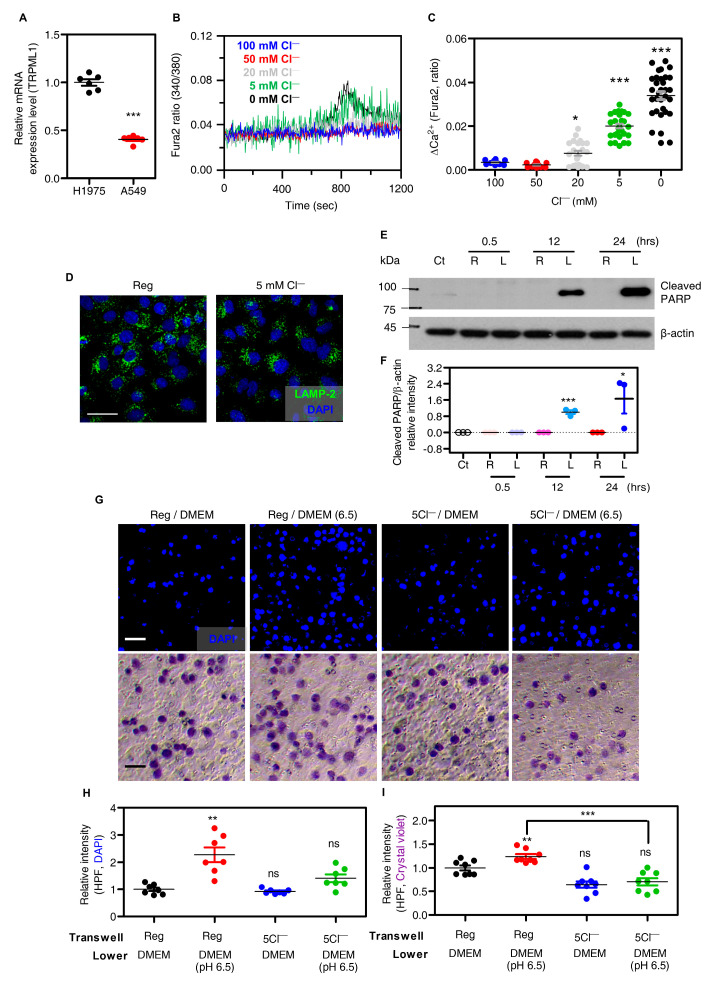
The similar effect of low Cl^−^ on other lung cancer cell A549. (**A**) The dot plots are presented as means ± SEMs of TRPML1 mRNA expression in H1975 and A549 cells (n = 6, *** *p* < 0.0001). (**B**) Changes in [Ca^2+^]_i_ in A549 cells with various range of Cl^−^ concentration from 0 to 100 mM. (**C**) The dot plots are presented as means ± SEMs of the relative changes of [Ca^2+^]_i_ spikes (ΔCa^2+^, n = 12, n = 13, n = 22, n = 28, n = 32, * *p* < 0.05, *** *p* < 0.0001). (**D**) Images of immunofluorescence staining of LAMP−2 (green) and DAPI (blue) in A549 cells which incubated with Reg and 5Cl^−^ solutions for 30 min. The scale (white) represents 25 μm. (**E**) Western blotting analysis of cleaved PARP and β-actin in A549 cells incubated with Reg (R) and 5Cl^−^ solution (L) during the indicated time. (**F**) The dot plots are presented as means ± SEMs of the protein band normalized to β-actin (n = 3, * *p* < 0.05, *** *p* < 0.001). (**G**) Images of transwell migration assay with DAPI (blue) or crystal violet (purple) which were incubated under the indicated conditions in A549 cells. The scale (white for DAPI and black for crystal violet) represents 50 μm. (**H**,**I**) The dot plots are presented as means ± SEMs of the relative intensity of DAPI and crystal violet (n = 4, ** *p* < 0.01, *** *p* < 0.001, ns; non-significance).

We performed most experiments on the epidermal growth factor receptor (EGFR) mutant cell line H1975. We confirmed the presence of EGFR wild type cell lines A549 in non-small cell lung cancer. Expressions of TRPML1 mRNA and protein in H1975 cells were higher than those in another non-small cell lung cancer cell lines, A549 (Figure 7A). The decreased [Cl^−^]_e_, ranging from 0 to 100 mM, induced no changes in [Ca^2+^]_i_ increase in A549 cells, and less than 5 mM [Cl^−^]_e_ induced a delayed small peak of Ca^2+^ signaling compared to that of H1975 cells (Figure 7B,C). Lysosomal repositioning (LAMP-2 fluorescence) by low Cl^−^ stimulation was also rarely observed in A549 cells (Figure 7D). We determined whether low Cl^−^ levels mediated apoptotic signal in A549 cells. Long-term application revealed the enhanced cleaved PARP expression (Figure 7E,F). We then determined whether the relatively low lysosomal response of A549 cells compared to that of H1975 cells, induced by low Cl^−^, induces cellular migration. Migration assays using DAPI and crystal violet revealed that low Cl^−^ stimulation reduced A549 migration, as shown in H1975 cells (Figure 7G–I). Although the degree of lysosomal Ca^2+^ release differed, stimulation with low Cl^−^ also inhibited A549 migration.

## 4. Discussion

Here, we addressed the changes in Cl^−^ concentration and modulated TRPML1-mediated Ca^2+^ release and TRPML1 trafficking. Depleted or reduced Cl^−^ mediated acute lysosomal Ca^2+^ release through TRPML1 and long-term exposure to reduced Cl^−^ enhanced apoptosis through lysosomal Ca^2+^ store depletion. The two GXXXP motifs of TRPML1—which we termed CBM1 and CBM2—in the lysosomal lumen sensed lysosomal Cl^−^ concentration, and its mutation reduced TFEB translocation and cellular migration.

TRPML1 is a nonselective cation channel involved in various cellular functions in migration and lysosomal biogenesis through endo/lysosomal Ca^2+^ release [38,39,40]. It is well established that TRPML1-mediated Ca^2+^ release is involved in lysosomal fusion and retrograde movement to the juxtanuclear region [4,41]. TRPML1 modulation has been addressed in the stimulation of phosphatidylinositol (3,5) bisphosphate (PI(3,5)P2) [42], acidic pH [43,44], reactive oxygen species [45], and the *inositol polyphosphoate-5-phosphatase* (*OCRL*) gene [46].

Cl^−^ is a homeostatic and signaling ion [47]; as a signaling ion, reduction of Cl^−^ concentration enhanced endosome Ca^2+^ channel activity in early endosome [48]. Our previous study addressed Cl^−^ as a modulating factor for NBCe1-B activity [24]. Additionally, the Cl^−^ sensing role potentially possessed by GXXXP motifs predicted various Cl^−^ transporters and channels, such as the Slc26 family, Na^+^-K^+^-Cl^−^ cotransporter, Na^+^-Cl^−^ cotransporter, and cystic fibrosis transmembrane conductance regulator [24]. Modulation of high lysosomal Cl^−^ has been suggested to maintain lysosomal function [3]. However, whether lysosomal Cl^−^ affects TRPML1-mediated Ca^2+^ release remains relatively unknown. Reduction of Cl^−^ results in enhanced lysosomal H^+^ ions through the involvement of Ca^2+^/H^+^ exchanger, V-type ATPase, and CLC-7 [49]. Thus, low Cl^−^ induces H^+^ influx into the lysosomal lumen and subsequent Ca^2+^ release through TRPML1, likely due to the secondary effect of lysosomal acidification [27]. However, the reduced Ca^2+^ response by low Cl^−^ in two GXXXP mutants of TRPML1, without changes in lysosomal vesicle pH, indicate that reduced Cl^−^ itself could be considered as a TRPML1 activator. Moreover, we addressed the predicted Cl^−^-sensing motif in TPCs, including TRPML1. TPCs and TRPML1 have different functions in endo/lysosomes [40]. Thus, the involvement of TPCs in this process was negligible. The difference in the Cl^−^ sensing position may provide diverse functions between the two types of lysosomal Ca^2+^ channels: TRPML1 and TPCs. Furthermore, in agreement with other cellular stresses, such as nutrient starvation or reactive oxygen species to activate TRPML1, low Cl^−^ can be a regulatory signaling factor of TRPML1 activity and lysosomal function.

**Figure 8:**
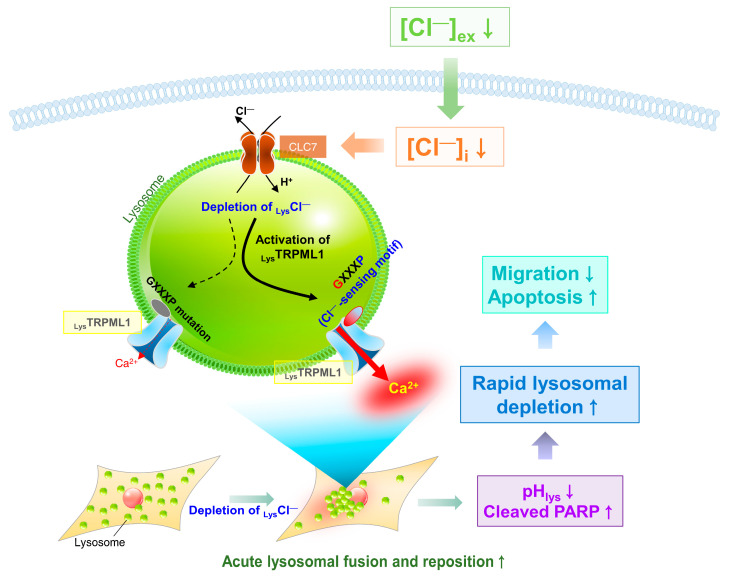
Schematic illustration of role of Cl^−^-dependent lysosomal Ca^2+^ release through TRPML1. Schematic illustration of the mechanism of Cl^−^ mediated lysosomal function, including TRPML1 activation and lysosomal reposition. The GXXXP motifs of TRPML1 sense the lysosomal Cl^−^ concentration. The short period of low Cl^−^ mediated Ca^2+^ drives the juxtanuclear clustering of lysosomes. Long-term exposure to low Cl^−^ mediates rapid lysosomal depletion, decreases migration, and increases apoptosis.

The role of Cl^−^ as a signaling ion in lysosomes is unknown. Notably, we provided evidence that the luminal GXXXP motif of TRPML1 sensed changes in intracellular Cl^−^ acutely (less than 30 min), and subsequent TRPML1-mediated Ca^2+^ release allows lysosomal movement (Figure 8). Lysosomal repositioning is regulated by various signaling pathways, such as nutrient deficiency [50,51,52], pathologic infection by pathogens [53,54,55], and cellular changes or stresses [56,57,58,59]. We observed dynamic phenomena of lysosomal movement over a short period of time. Without nutrient starvation, perhaps before the recognition of starvation, depleted Cl^−^ is sufficient to drive dynamic lysosomal trafficking with Ca^2+^ involvement. In addition, low Cl^−^-mediated acute lysosomal Ca^2+^ release was used in the juxtanuclear clustering of lysosomes, which is a hallmark of lysosome activation [16]. Acidification of lysosomes and prolonged low Cl^−^ conditions reduced migration and enhanced apoptosis.

Lysosomal Ca^2+^ release is known to play a crucial role in cellular migration and progression [5], and the lysosomal Ca^2+^ channel TRPML1 provides a Ca^2+^ source to induce autophagic vesicles [60]. Although it is clear that there is a role for TRPML1 in cell survival and expansion, interestingly, enriched lysosomal Cl^−^ levels are crucial, and reduced Cl^−^ level is acutely monitored by TRPML1 through the GXXXP motif. Its low Cl^−^-mediated Ca^2+^ release and lysosomal trafficking may provide a survival strategy against Cl–depletion within a short period of time. In recent studies, it other Cl^−^ channels have been demonstrated, such as leucine-rich repeat-containing protein (LRRC)8 and ceroid lipofuscinosis (CLN)7. LRRC8 is a volume-regulated anion channel which senses cellular osmolality [61,62,63]. Although deletion of LRRC8 increases Hela cell death through hypotonicity, our data showed that low Cl^−^ was not associated with osmolality, as shown in Figure 1G. Defects of CLN7 cause neurodegeneration through mutation of *major facilitator superfamily domain containing 8* (*MFSD8)* gene [64]. Low Cl^−^ has the potential to mediate CLN7 because CLN7 is activated by acidic pH [65]. However, CLN7 currents are not generated in low Cl^−^ condition [65].

Lysosomal Ca^2+^ release by TRPML1 activating signals, such as low Cl^−^ in the current study and subsequent notable changes in the lysosome-mediated mechanism, can be highly potent in regulating cellular fate. The mechanism of dysregulated lysosomal activity in several lysosomal diseases remains unclear. Lysosomal Cl^−^ has evolved from obscurity to a topic progress in the fascinating field of lysosome biogenesis. In addition, this study attempts to explain that low Cl^−^ stimulation could function as a direct and acute tool by which to verify lysosomal Ca^2+^ storage. On the other hand, lysosomal activity in cancer has been considered contradictory because of its diverse effects [59,66,67]. Although we faced the direct measurement of lysosomal TRPML1 current to verify our hypothesis, measurement of the lysosomal current required an enlarged lysosome via vacuolin-1 treatment [68]. It is known that regulation of lysosomal vacuoles is driven by TRPML1-mediated Ca^2+^ release [69]. Accordingly, to identify the physiological role of low Cl^−^-driven TRPML1 activation, we performed the analysis of lysosomal changes in the exposure of low Cl^−^ on intact cells without the artificial enlargement of lysosomes.

As we addressed in this study, the low Cl^−^ approach induced the depletion of the lysosomal pool, and subsequent excessive lysosomal activity via acute lysosomal Ca^2+^ release through TRPML1 dysregulated lysosomal biogenesis brought about destruction of the terminal recycling center. It thus appears that Cl^−^ depletion targeting approaches, such as that of the putative lysosomal Cl^−^ quencher, are the starting point for improved therapeutic strategies for the lysosomal targets of cancer and the dysfunction of lysosome-mediated diseases.

## Figures and Tables

**Table 1:** Composition of solutions.

Composition (Regular Solution, Reg)				Concentration (mM)	
Sodium chloride (NaCl)				140	
HEPES				10	
Glucose				10	
Potassium chloride (KCl)				5	
Magnesium chloride (MgCl_2_)				1	
Calcium chloride (CaCl_2_) (not added in C/F)				1	
pH 7.4					
310 mOsm (adjusted with mannitol)					
**Composition** **(chloride solutions)**	**Concentration (mM)**				
	100 Cl^−^	50 Cl^−^	20 Cl^−^	5 Cl^−^	0 Cl^−^
Sodium chloride (NaCl)	100	50	20	5	0
Sodium gluconate	40	90	120	135	140
HEPES	10				
Glucose	2				
Potassium gluconate	5				
Magnesium sulfoxide (MgSO_4_)	1				
Calcium gluconate (not added in C/F)	1				
pH 7.4					
310 mOsm (adjusted with mannitol)					

The Reg was composed of NaCl, HEPES, glucose, KCl, MgCl_2_, and CaCl_2_ in condition of pH7.4 and 310 mOsm. The Cl^−^ concentration of chloride solution was adjusted by the mass of NaCl and Na^+^ concentration was supplemented with sodium gluconate.

## Data Availability

The datasets analyzed during the current study are available from the corresponding author on reasonable request.

## Supplementary Materials

The following supporting information can be downloaded at: https://www.mdpi.com/article/10.3390/cells12141835/s1, Figure S1: The pH calibration curves of BCECF and pHRodo; Figure S2: The effect of low Cl^−^-mediated Ca^2+^ signaling on ER Ca^2+^ release; Figure S3: The modulation of TPC expression and TRPM2 GXXXP motif have no effect on low Cl^−^-induced Ca^2+^ signaling; Figure S4: The effect of TRPML1 clones on LAMP2 location and vesicle pH; Figure S5: The effect of siTRPML1 on C/F 5 mM Cl^−^ solution-mediated [Ca^2+^]_i_ signaling; Figure S6: Transwell migration assay in presence of ML-SA1 or overexpressed all types of TPRML1 clones; Figure S7: Western blotting analysis with siCLC3, siCLC7, and siTRPML1.
