# Supplementary material for: Modulation of Lysosomal Cl− Mediates Migration and Apoptosis through the TRPML1 as a Lysosomal Cl− Sensor
Source: Cells. 2023 Jul 12;12(14):1835. doi: 10.3390/cells12141835 (PMC10378694; doi:10.3390/cells12141835)
Supplement: Supplementary file 1 [file cells-12-01835-s001.zip › cells-2434877-supplementary.pdf]

Modulation of lysosomal Cl^- mediates migration and apoptosis through the TRPML1 as a lysosomal Cl^- sensor

Cells

Dongun Lee and Jeong Hee Hong*

Department of Health Sciences and Technology, Lee Gil Ya Cancer and Diabetes Institute, GAIHST, Gachon University, 155 Getbeolro, Yeonsu-gu, Incheon, 21999, South Korea

* Correspondence to JHH (minicleo@gachon.ac.kr)

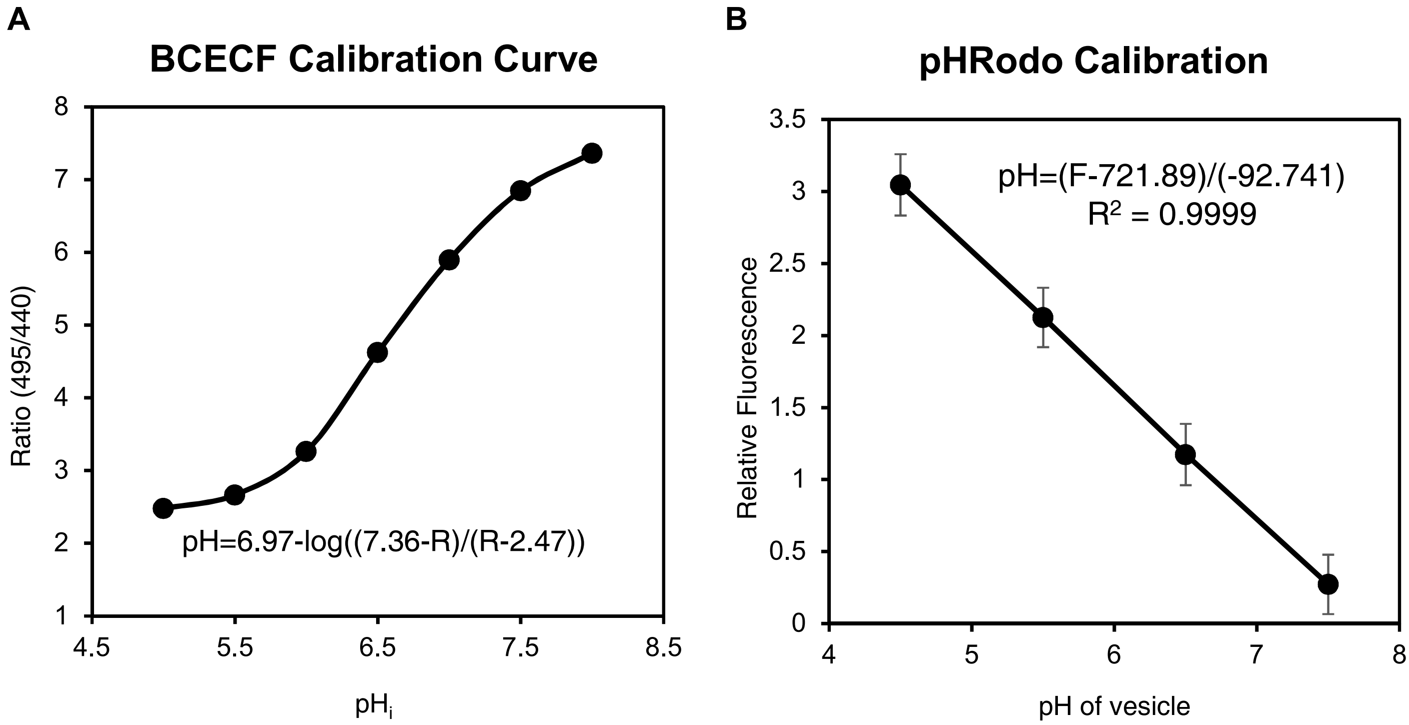

Supplementary Figure S1

The pH calibration curves of BCECF and pHRodo

(A) The pH calibration curves for BCECF-loaded H1975 cells at pH 5.5, 6.0, 6.5, 7.0, 7.5, 8.0, and 8.5. The regression curve was pH=6.97-log((7.36-R)/(R-2.47)), where R is the ratio of BCECF fluorescence. **(B)** The pH calibration curve for pHRodo-loaded H1975 cells at pH 4.5, 5.5, 6.5, and 7.5. Regression curves are pH =(F-721.89)/(-92.741), F: intensity of pHRodo fluorescence.

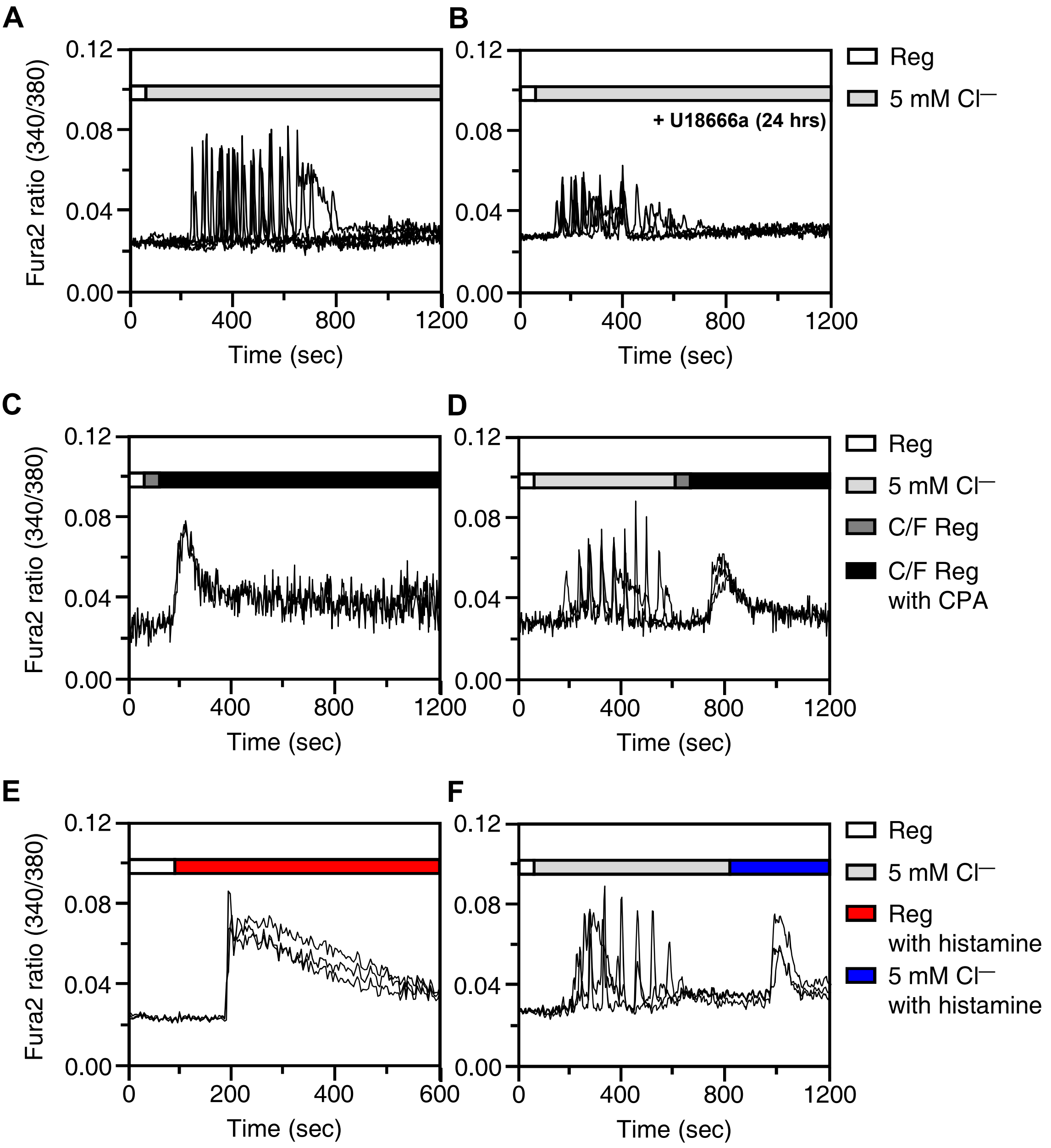

Supplementary Figure S2

The effect of low Cl⁻-mediated Ca^{2+} signaling on ER Ca^{2+} release

(A, B) Changes in $[Ca^{2+}]_i$ in H1975 cells with 5 mM Cl⁻-induced Ca^{2+} signaling in the absence of U18666a (4 μg/mL, 24 hrs) or in the presence of U18666a. **(C, D)** Changes in $[Ca^{2+}]_i$ in H1975 cells in CPA treatment (C) and 5 mM Cl⁻-induced Ca^{2+} signaling with subsequent treatment of CPA (D). Ca^{2+} free concentration is defined as C/F. **(E, F)** Changes in $[Ca^{2+}]_i$ in H1975 cells in histamine treatment (E) and 5 mM Cl⁻-induced Ca^{2+} signaling with subsequent treatment of histamine (F).

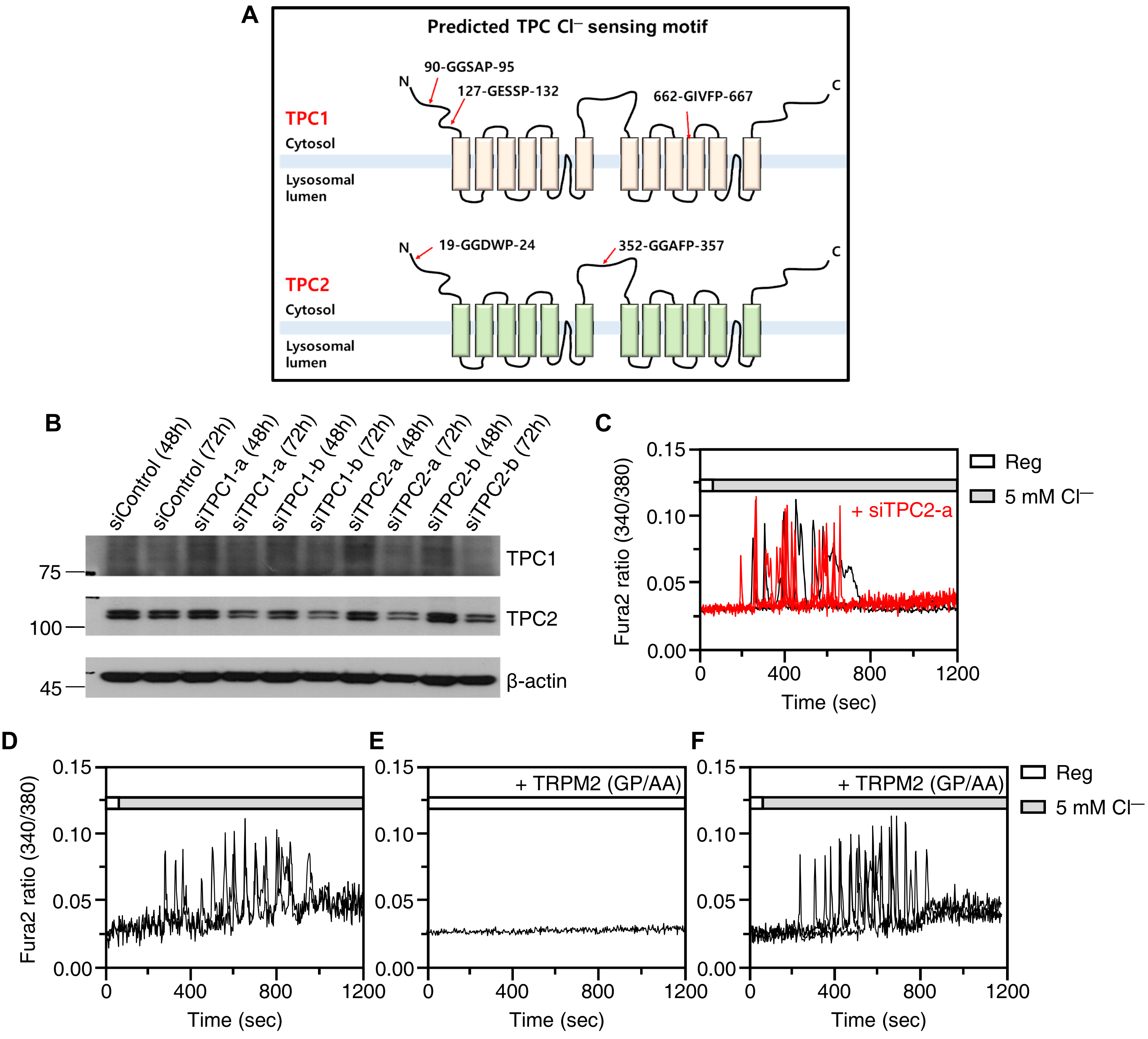

Supplementary Figure S3

The modulation of TPC expression and TRPM2 GXXXP motif have no effect on low Cl⁻-induced Ca^{2+} signaling

(A) Schematic illustration of TPCs structure marked with GXXXP motifs. **(B)** Western blotting analysis of TPC1 and TPC2 in siTPC1- or siTPC2-transfected H1975 cells. **(C)** Changes in $[Ca^{2+}]_i$ in siTPC2-a-transfected H1975 cells with 5 mM Cl⁻ solution. **(D-F)** Changes in $[Ca^{2+}]_i$ in H1975 cells with 5 mM Cl⁻ solution (D), Reg with TRPM2-GP/AA (E), 5 mM Cl⁻ with TRPM2-GP/AA (F).

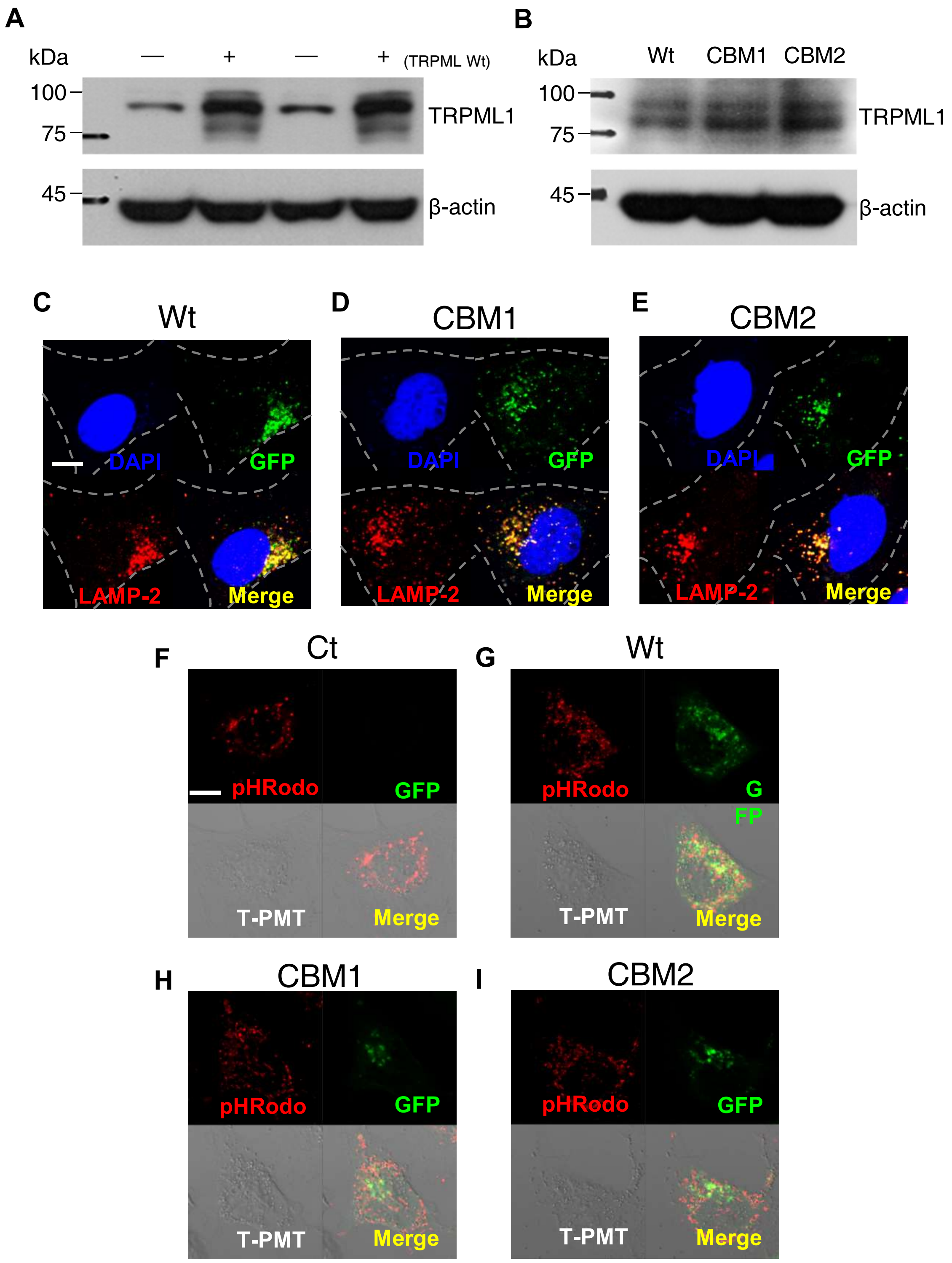

Supplementary Figure S4

The effect of TRPML1 clones on LAMP2 location and vesicle pH

(A) Western blotting analysis of TRPML1 protein in H.T cells transfected with TRPML1 Wt. **(B)** Western blotting analysis of TRPML1 protein in H1975 cells transfected with TRPML1 Wt, CBM1, and CBM2. **(C-E)** Confocal images of immunofluorescence staining of LAMP-2 (red) and DAPI (blue) which transfected with GFP-tagged TRPML1 Wt, CBM1, and CBM2 (green). The scale (white) represents 10 μm. **(F-I)** Confocal images of immunofluorescence staining of pHRodo (red) in control (F) and cells transfected with GFP-tagged TRPML1 Wt (G), CBM1 (H), or CBM2 (I). The scale bar (white) is 10 μm.

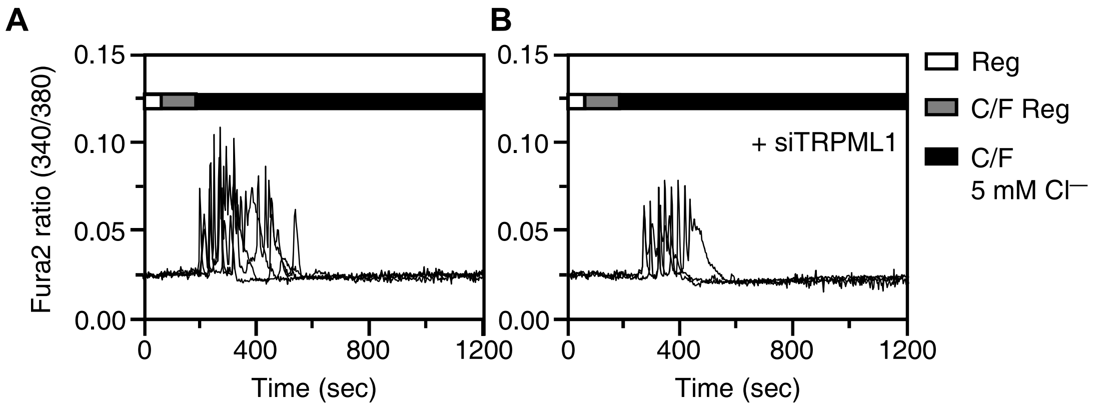

Supplementary Figure S5

The effect of siTRPML1 on C/F 5 mM Cl⁻ solution-mediated $[Ca^{2+}]_i$ signaling

(A, B) Changes in $[Ca^{2+}]_i$ in H1975 cells (A) and with knock-down of TRPML1 (B).

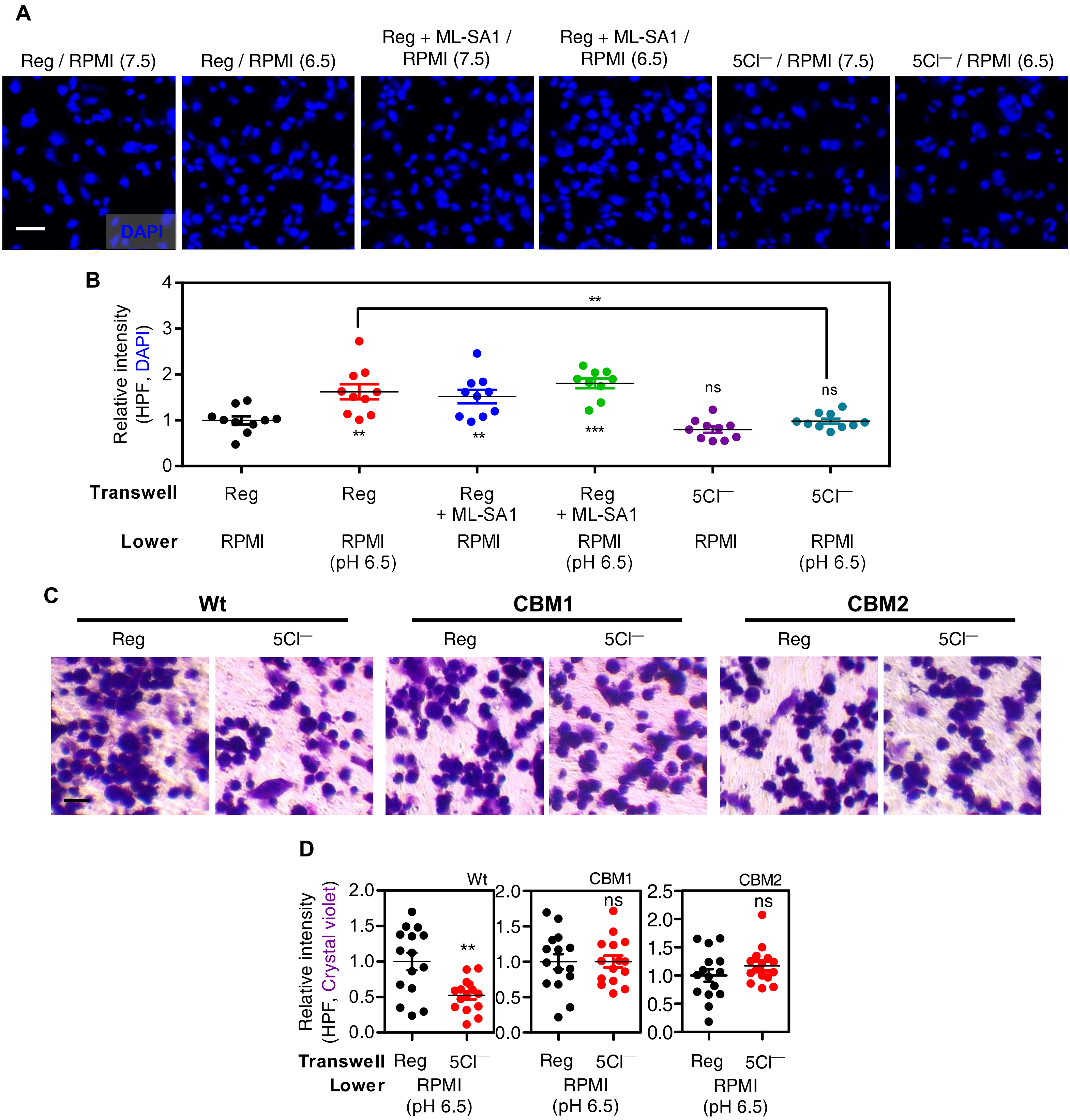

Supplementary Figure S6

Transwell migration assay in presence of ML-SA1 or overexpressed all types of TPRML1 clones

(A) Images of transwell migration assay with DAPI (blue) which incubated for under the indicated conditions. **(B)** The dot plots are presented as means ± SEMs of the relative intensity of DAPI (**p < 0.01, ns; non-significance). **(C)** Images of transwell migration assay with crystal violet (purple) in TRPML1, CBM1, and CBM2-transfected cells with the indicated conditions. **(D)** The dot plots are presented as means ± SEMs of the relative intensity of crystal violet (*p < 0.05).

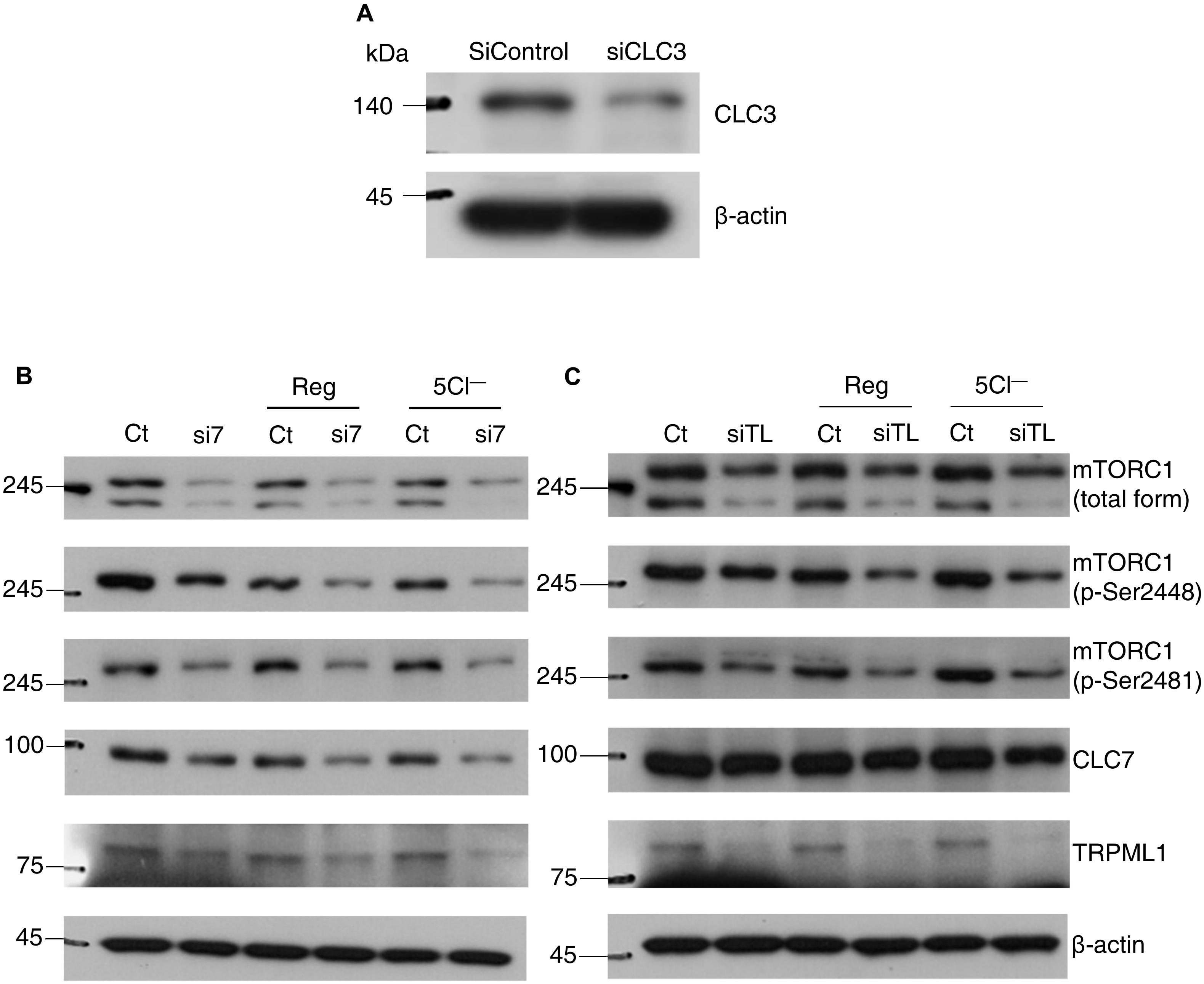

Supplementary Figure S7

Western blotting analysis with siCLC3, siCLC7 and siTRPML1

(A) Western blotting analysis of CLC3 transfected with siRNA-CLC3 (siCLC3). **(B)** Western blotting analysis of mTORC1, two types of phosphorylated mTORC1 (ser-2448 and ser-2481), CLC7 and TRPML1 transfected with siRNA-CLC7 (si7) in presence of Reg or 5 mM Cl^- solution for 30 min. **(C)** Western blotting analysis of mTORC1, phosphorylated mTORC1 (ser-2448 and ser-2481), CLC7 and TRPML1 transfected with siRNA-TRPML1 (siTL) in presence of Reg or 5 mM Cl^- solution for 30 min.
